# Separation of Microplastic Particles from Sewage Sludge Extracts Using Magnetic Seeded Filtration

**DOI:** 10.1016/j.wroa.2022.100155

**Published:** 2022-09-13

**Authors:** Frank Rhein, Hermann Nirschl, Ralf Kaegi

**Affiliations:** a Karlsruhe Institute of Technology (KIT), Institute of Mechanical Process Engineering and Mechanics Strasse am Forum 8, Karlsruhe 76131 Germany; b Eawag, Ueberlandstrasse 133, Dübendorf 8600 Switzerland

**Keywords:** Microplastics, Cellulose, Magnetic Separation, Extraction, Sewage Sludge, Environmental Samples

## Abstract

Microplastic particles (MP) are efficiently retained in wastewater treatment plants and enriched in sewage sludge. For monitoring MP contents in wastewater systems, sewage sludge is thus well suited, but also requires an isolation of MP from the sludge matrix, as other sewage sludge components may interfere with the MP identification and quantification. Although organic matter in sludge samples can be removed through acid and enzymatic digestion procedures, cellulose - mainly from toilet paper - remains in the digests, due to its high chemical resistivity and similar density to MP. We apply the separation concept of magnetic seeded filtration to isolate MP through selective hetero-agglomeration with magnetic seed particles. MP and cellulose differ in their hydrophobic properties and we investigate to what extent these differences can be exploited to selectively form MP-magnetite hetero-agglomerates in the presence of cellulose. These hetero-agglomerates are subsequently separated using a magnet. Five MP types (Polyethylene terephthalate (PET), polypropylene (PP), low density polyethylene (LDPE), polyvinyl chloride (PVC) and polystyrene (PS)) and cellulose particles were mixed in different combinations with both hydrophilic and hydrophobic (silanized) magnetite particles. PET, PP, LDPE and PS only poorly agglomerated with pristine (hydrophilic) magnetite, but efficiently formed hetero-agglomerates with hydrophobic magnetite and were successfully removed from suspensions (80−100%). PVC agglomerated more efficiently with pristine than with hydrophobic magnetite and cellulose only agglomerated to a limited extent with either hydrophilic or hydrophobic magnetite, resulting in a high process selectivity. Results from experiments conducted at different ionic strengths and with hydrophilic and hydrophobic magnetite suggests that the agglomeration process was dominated by hydrophobic interactions. Enzymatic and oxidative treatment of the MP only marginally affected the separation efficiencies and (treated) MP spiked to sewage sludge extracts were successfully recovered using magnetic seeded filtration.

## Highlights

• Magnetic seeded filtration can efficiently separate microplastics from cellulose.
• Enzymatic and oxidative treatments only marginally affect separation efficiencies.
• Hydrophobic interactions govern the hetero-agglomeration process.
• The agglomeration behavior of PVC differs from that of other microplastics.

## Graphical abstract

**Figure:**
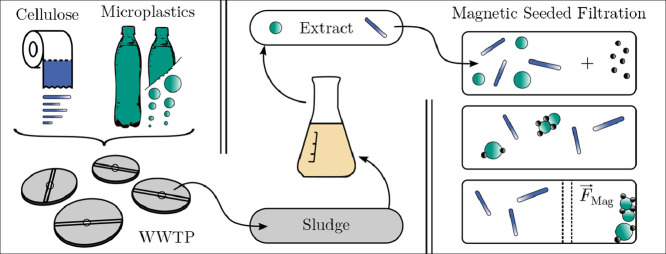

## Nomenclature

N: Number concentration [$m^{-3}$]
β: Collision frequency [$m^{-3}s^{-1}$]
α: Collision efficiency [ - ]
r: Particle radius [m]
$\bar{G}$: Mean shear rate [$s^{-1}$]
E: Interaction energy [${\mathrm{kgm}}^{2}s^{-2}$]
$k_{B}$: Boltzmann constant [${\mathrm{kgm}}^{2}s^{-2}K^{-1}$]
$\vartheta_{A}$: Absolute temperature [K]
$F_{Mag}$: Magnetic force [N]
$\mu_{0}$: Permeability constant of vacuum [${\mathrm{NA}}^{-2}$]
M: Magnetization [${\mathrm{Am}}^{-1}$]
H: Magnetic field strength [${\mathrm{Am}}^{-1}$]
f: Frequency$\left[s^{-1}\right]$
X: Mass fraction[−]
x: Particle diameter[m]

## 1 Introduction

The superior properties of plastic materials, such as light weight, moldability and durability combined with low-cost production have lead to an enormous increase in the annual production of plastics since the start of the mass production in the late 60-ties and to its accumulation in the environment (Andrady, 2017; Birch et al., 2020; Geyer et al., 2017; Jambeck et al., 2015; MacLeod et al., 2021; Ostle et al., 2019). The ongoing fragmentation of larger plastic items due to mechanical and photochemical weathering results in the formation of microplastic particles (MP) referring to particulate, synthetic polymers with diameters of less than5mm (e.g., Hartmann et al., 2019; Thompson et al., 2004), which have been reported from regions all over the globe.

The identification and quantification of MP in various matrices is currently achieved either on an individual particle level or based on total MP quantities in a sample. In the fist case, vibrational spectroscopic measurements (e.g., Fourier transform - infrared (FT-IR) or RAMAN) are conducted at spatial resolutions of≈1−10μm (Anger et al., 2018; Ivleva, 2021; Pico et al., 2019; Primpke et al., 2020 and in the second case, mass spectroscopic methods, such as pyrolysis gas chromatography (GC), mass spectrometry (MS) (Dierkes et al., 2019; Funck et al., 2020; Okoffo et al., 2020) or thermal extraction desorption (TED) GC-MS (Duemichen et al., 2019; Eisentraut et al., 2018; Yakovenko et al., 2020) are used. In both cases, however, interference with matrix compounds challenge the analytical techniques and several sample preparation techniques have been developed and tailored for specific matrices (Blaesing and Amelung, 2018; Han et al., 2019; He et al., 2018; Junhao et al., 2021; Müller et al., 2021; Stock et al., 2019.

Wastewater treatment plants (WWTP) play a pivotal role in removing MP from municipal and industrial wastewater. Reported removal efficiencies are mostly above80−90% (reviewed for example by Ali et al. (2021) and Gatidou et al. (2019)) and most recent studies report that state of the art WWTP even exceed these percentages considerably (Rasmussen et al., 2021). This leads to an accumulation of MP in the sewage sludge. MP contents in sewage sludge matrices, therefore, received considerable attention and several extraction procedures tailored for such matrices have been suggested (Hurley et al., 2018; Sujathan et al., 2017). Philipp et al., 2022 showed that easily degradable organic materials can be mineralized using a combination of oxidative and enzymatic digestions and inorganic (sand) particles can be separated from the MP based on density differences. However, cellulose materials remain in the digests due to similar size, density and chemical resistance of cellulose particles and MP.

As density separation and selective (enzymatic) digestions both failed to separate cellulose from MP, alternative separation methods have to be considered. Thereby, magnetic seeded filtration (MSF) seems very promising, which is also supported by initial results published by Grbic et al. (2019). Magnetic seeded filtration (MSF), also referred to as magnetic flocculation (Tsouris et al., 1995) and magnetic adsorption (Franzreb, 2020) is a solid-liquid separation process, which has been used in various applications (Franzreb, 2020). The approach is based on the selective hetero-agglomeration between magnetic seed particles and the (nonmagnetic) target particles. The agglomerates containing the magnetic seed particles are separated by magnetic separation, whereas permanent magnets can be used. MSF can also be applied to non-particulate products, in which case the target analytes are adsorbed to the surface of the magnetic seed particles. This separation process has for example been applied for harvesting microalgae (Wang et al., 2015), removing oil leaks (Mirshahghassemi et al., 2017), and purifying proteins (Ebeler et al., 2019; Franzreb et al., 2006). Furthermore, MSF was applied to remove phosphates (Du et al., 2019) and fine particle matter (Chin et al., 2006; Gray et al., 1994; Wan et al., 2011) from wastewater and natural organic matter (NOM) during pre-treatment of drinking water production on an industrial scale (Drikas et al., 2011). Employing MSF for the separation of micro- and nanoplastics is currently extensively researched by multiple groups, with ever increasing possibilities regarding the magnetic seed material (Groppe et al., 2022; Misra et al., 2020; Shi et al., 2022a). Recent studies show that MSF is capable of achieving high separation efficiencies for microplastics Rhein et al. (2019b) under laboratory conditions and identified benefits included the high material specific selectivity and the applicability in dilute suspensions (Franzreb, 2020; Rhein et al., 2019b). However, as MSF has not yet been widely applied in large-scale applications, no general estimations on cost effectiveness exists, yet. Thus, MSF is currently a promising approach for separating MP from complex (environmental) samples in the laboratory i.e. on small scales (Grbic et al., 2019; Shi et al., 2022b; Zhang et al., 2021).

In this work, we investigated the potential of MSF to separate MP from cellulose in sewage sludge extracts. We first determined the separation efficiencies in two component systems consisting of magnetic seed particles and different MP types or cellulose particles. Both hydrophilic and hydrophobic magnetic seed particles were used to assess the impact of hydrophobic interactions. Building on these experiments, each individual MP type was mixed with cellulose and then separated using magnetic particles (3-component system) to determine the selectivity of the MSF process and to investigate whether possible interactions between cellulose particles and MP affected the selectivity. As a proof of principle, different types MP were added to and separated from a sewage sludge extract, containing large amounts of cellulose particles. This work, therefore, aims at the possible application of MSF for analytical purposes rather than on an assessment of its suitability for large-scale applications in e.g., potable water treatment.

## 2 Materials & Methods

### 2.1 Magnetic seeded filtration theory

The key process, defining the efficiency of MSF is the hetero-agglomeration between magnetic and (non-magnetic) target particles. The agglomeration kinetic between two particlesi andj resulting in agglomerateij can be expressed according to relation 1 (Elimelech, 1998).

(1) $$\frac{dN_{\mathrm{ij}}}{dt}\propto N_{i}N_{j}\beta_{i,j}\alpha_{i,j}$$

$N_{i}$ and$N_{j}$ describe the number concentrations of agglomerating particles. To form an agglomerate, two particles must collide, which is quantified by the collision frequencyβ. In this study, agglomeration is governed by fluid flow rather than diffusion due to large particle diameters andβ can therefore be approximated according to Eq. 2 (Chin et al., 1998; Elimelech, 1998; Han and Lawler, 1992). The collision frequency is determined by the particle radiir and mean shear rate in the system$\bar{G}$.

(2) $$\beta_{i,j}=\frac{4}{3}\left(r_{i}+r_{j}\right){}^{3}\bar{G}$$

Upon collision, two particles must “stick” together, the probability of which is expressed through the collision efficiencyα in relation 1. The calculation ofα is non-trivial, especially in the shear-controlled regime, as it requires calculation of particle trajectories under consideration of the flow conditions (Elimelech, 1998; Han and Lawler, 1992). The relation betweenα and other relevant parameters is given by Reerink and Overbeek (1954) and Selomulya et al. (2003).

(3) $$\alpha_{i,j}\propto \underset{\left(a\right)}{\underset{?}{\exp\left(-C_{1}\left(1-\frac{r_{i}}{r_{j}}\right)\right){\left(r_{i}r_{j}\right)}^{-C_{2}}\;}}\underset{\left(b\right)}{\underset{?}{\exp\left(-\frac{E_{\Sigma ,\max}}{k_{B}\vartheta_{A}}\right)}}\;$$

Factor (a) describes the size dependency ofα according to Selomulya et al. (2003) and Soos et al. (2007) with empirical constants$C_{1}$ and$C_{2}$: For increasing particle size as well as decreasing particle size ratio$r_{i}/r_{j}\ll 1$, the collision efficiency and therefore the probability of an agglomeration is reduced due to increasing inertia of the particles. Factor (b) is an approximation for the maximum collision efficiency of the diffusion-controlled regime (Reerink and Overbeek, 1954) and is governed by the particle-particle interaction energyE. In the traditional DLVO approach (Derjaguin, 1954), the attractive van der Waals interactions and (mostly) repulsive electrostatic interactions are considered, whereas this work further requires the inclusion of attractive hydrophobic interactions. For a more detailed description, the reader is referred to Christenson and Claesson (2001), Elimelech (1998), Israelachvili (2011) and to SI.1.

After the hetero-agglomerates are formed, they are separated on the basis of their newly gained magnetic properties. The magnetic force given in Eq. 4 needs to overcome the drag of the hetero-agglomerates. It is primarily dependent on the gradient of the magnetic field strength∇H, the magnetizationM and partial volume$V_{M}$ of the magnetic component. It is referred to Svoboda (2004) for a more profound description of magnetic separation and the relevant material properties.

(4) $$F_{Mag}=\mu_{0}V_{M}M\nabla H$$

After separation, the agglomerates can be further processed to recycle the magnetic seed material and also recover the separated non-magnetic fraction. Agglomerates are either broken up through mechanical stress, or exposed to high temperatures or solvents to extract the non-magnetic component. A previous study (Rhein et al., 2021) showed high recovery rates and low performance losses over the course of multiple separation cycles for three different recycling approaches.

### 2.2 Preparation of MP fragments

Polyethylene terephthalate (PET), polypropylene (PP), low-density polyethylene (LDPE) and polystyrene (PS) beads were purchased from Goodfellow GmbH (Germany) with5mm diameter. To generate MP fragments of smaller sizes (x∼100μm),5g of the respective beads were ground in a ball mill (M400, Retsch GmbH, Germany) largely following the protocol of Seghers et al. (2021). To cool the35mL stainless steel buckets containing both the milling ball with a diameter of20mm as well as the MP beads to cryogenic temperatures, they were put in liquid nitrogen for10min. The materials were ground several times (PET: 5, PP: 16, LDPE: 15, PS: 5), until the desired size was reached. One grinding cycle consisted of1min of milling at$f=30\;s^{-1}$ and1.5min of cooling in liquid nitrogen.

### 2.3 Silanization of magnetite

Hydrophobic magnetite was obtained through silanization of magnetite powder (Kremer Pigments, Germany) with hexadecyltrimethoxysilane (HDTMS,>85% technical grade, Sigma-Aldrich), mainly following the procedures described by Frickel et al. (2010), Grbic et al. (2019) and Ji et al. (2017), which vary in solvent, the amount of added water (if any) and the amount of added HDTMS per surface area of particles. A particle surface specific HDTMS concentration of$4\times 10^{-4}\;\frac{\mathrm{mol}}{m^{2}}$ ensures sufficient excess to guarantee complete silanization of the magnetite particles. Assuming perfect spheres and neglecting particle porosity, the required amount of HDTMS can be calculated for different magnetite particle sizes (see SI.2 for more information).

Fourg of magnetite particles were added to a50mL plastic tube together with36mL of analytical grade ethanol (>99.9%, EMSURE, Merck, Germany),4mL of nanopure water and0.85mL of HDTMS. The suspension was mixed in an overhead shaker (Reax 2, Heidolph Instruments, Germany) for24h. The silanized magnetite particles were separated with a permanent magnet and the remaining liquid was discarded. Following this, the magnetite particles were washed three times with30mL of ethanol (technical grade) with magnetic separation of the magnetite particles between each washing step. The washed and silanized particles were dried at$40^{\circ }\;C$ for17h and particles from the same batch were used for all of the experiments. Throughout this work, hydrophobic magnetite is abbreviated with Mag-C16, indicating the alkyl chain length of HDTMS.

### 2.4 Treatment of MP

To extract MP particles from complex matrices, including sewage sludge, different protocols haven been suggested, most of them including combinations of enzymatic and oxidative digestion steps (see Section 1). This treatment, however, may induce changes of the surface properties of the MP, which may affect their agglomeration behavior during MSF. To assess these impacts, MP were exposed to enzymatic and oxidative treatment following the sample preparation protocol for sewage sludge described in Philipp et al. (2022). In brief, MP were first exposed to Fenton reagents by adding the MP to a mixture of10mL H${}_{2}$O${}_{2}$ (35%),5mL deionized (DI) water,1mL Fe(II)SO${}_{4}$ (7×H${}_{2}$O,2mM) and1mL protocatechuic acid (2mM). After a reaction time of1h on a horizontal shaker at room temperature and an additional12h at$40^{\circ }\;C$, MP were recovered by filtration and incubated with50mL phosphate citrate buffer (50mM,pH=5),0.5g cellulase (extracted from Aspergillus niger; Sigma-Aldrich, No. 22178) and10mg sodium azide at$40^{\circ }\;C$ for72h. MP were recovered again by filtration and rinsed with DI water. MP following these treatments are referred to as processed MP in the remainder of the manuscript. Attenuated total reflectance (ATR) - Fourier Transform (FT) - Infrared (IR) spectra recorded on the MP before and after the digestion steps were identical (see Fig. SI.3), demonstrating that the structure of the MP was not affected by the digestion procedure.

### 2.5 Analytical particle solvent extraction

The analytical particle solvent extraction (APSE) was performed following Sygusch and Rudolph (2021) to quantify the wettability of particulate systems. Twenty-fivemg of dry particle powder was added to5mL of ultrapure water in a15mL plastic tube and dispersed in an ultrasound bath for2min. Subsequently,5mL of 1-Octanol (analytical grade,>99.9%, Sigma-Aldrich) was added and the mixture was again dispersed for2min in an ultrasound bath. Then, the suspension was mixed in an overhead shaker (Reax 2, Heidolph Instruments, Germany) for20min. After mixing, the suspension was allowed to rest until a clear phase separation between octanol and water was apparent. Then,5mL of the top (octanol) phase were collected with a pipette, and the remaining aqueous fraction was decanted and the tube rinsed with ultrapure water. Both fractions were dried at$40^{\circ }\;C$ and weighted to determine the particle masses in the octanol and in the aqueous phase. The mass fraction in the octanol phase$X_{Octanol}$ was calculated according to Eq. 5 and serves as measure for hydrophobicity: Higher values of$X_{Octanol}$ indicate more hydrophobic particles as they tend to accumulate in the non-polar phase. All extraction experiments were performed in triplicate.

(5) $$X_{Octanol}=\frac{m_{Octanol}}{m_{Octanol}+m_{H_{2}O}}$$

The challenge of this procedure is the accumulation of particles at the liquid-liquid interface. This may either happen directly or due to sedimentation of particles in the octanol phase. The latter was remedied by decreasing resting time, however the APSE did not yield reliable results for MP, as most of the material was found at the liquid-liquid interface. The results of the APSE were used to both validate the silanization of the magnetite particles discussed in Section 2.3 and to characterize the behavior of the cellulose particles.

### 2.6 Particle properties

PET, PP, LDPE and PS particles were prepared through cryo milling as explained in Section 2.2, and polyvinyl chloride (PVC) particles were purchased in powder form. Pulp fibers (J. Rettenmaier & Söhne GmbH, Germany) commonly used as filter aids were used as surrogate for cellulose Bächle et al. (2021). Magnetite particles (Kremer Pigmente, Germany) were used as magnetic seed material and have been described in Rhein et al. (2019b). All particle types were investigated by scanning electron microscopy (SEM) and the resulting images, along with the operational conditions of the SEM are shown in Fig. SI.2. Besides revealing the fibrous shape of cellulose particles, distinct differences between the cryo-milled particles (PET, PP, LDPE, PS) and PVC are apparent: PVC particles exhibit smooth surfaces and spherical shapes, whereas the cryo milling process led to diverse and often flattened shapes with rugged particle surfaces. The cumulative size distributions of all particle types (MP, magnetite, cellulose), derived from static laser light scattering measurements (LS13 320 XR, Beckman Coulter) are shown in Fig. SI.5 The$x_{50}$ of all types of MP were within one log10 unit and ranged from150μm (PVC) to450μm (LDPE/PP). The cumulative size distribution of the cellulose particles was very similar to the cumulative size distributions of the MP, however, this needs to be interpreted carefully due to the fibrous shape of the cellulose particles. The$x_{50}$ of the magnetite particles was roughly two orders of magnitude smaller than both the MP and cellulose particles resulting in a higher collision probability due to higher number concentrations and a higher volume specific surface area compared to the MP, which enhances the hetero-agglomeration process.

The pristine magnetite particles were hydrophilic, but were made hydrophobic through the silanization process (Table 1). Cellulose fibres were hydrophilic. Strong accumulation of MP at the the liquid-liquid interface during the APSE process hampered an assessment of the hydrophobic properties of the MP and contact angle measurements on polymer films of the same material from the same manufacturer were performed in triplicate to assess the hydrophobicity of the MP. Contact angles were measured on films, as determining the contact angle of powders often yields unreliable results (Sygusch and Rudolph, 2021). All polymer films exhibit contact angles above$64{\;}^{\circ }$, with slight differences between the individual film types (Table 1). The properties of the pristine and the processed MP, the cellulose and the magnetite particles, along with the properties of the plastic films are summarized in Table 1.

**Table 1:** Properties of the particulate materials and of the polymer films (PET: Polyethylene terephthalate; PP: polypropylene; LDPE: low-density polyethylene, PVC: polyvinyl chloride, PS: polystyrene).

	Magnetic (M)	Non-magnetic (NM)					
Material	Magnetite (Fe${}_{3}$O${}_{4}$)	Cellulose (Pulp)	PET	PP	LDPE	PVC	PS
Manufacturer	Kremer Pigments	J. Rettenmaier & Söhne GmbH	Goodfellow	Goodfellow	Goodfellow	Goodfellow	Goodfellow
Density $\rho^{a}\left[\frac{\mathrm{kg}}{m^{3}}\right]$	5200	1500	1400	900	920	1400	1100
$x_{10,3}^{b}\left[\mu m\right]$	2.1	110	150	230	210	110	130
$x_{50,3}^{b}\left[\mu m\right]$	7.2	310	310	450	450	150	280
$x_{90,3}^{b}\left[\mu m\right]$	21	590	490	650	1100	210	490
Contact angle $\theta \left[{}^{\circ }\right]$ pristine	${-}^{c}$	${-}^{c}$	$77\pm 0.7^{d}$	$100\pm 3.4^{d}$	$97\pm 0.6^{d}$	$85\pm 1.3^{d}$	$90\pm 3.2^{d}$
Contact angle $\theta \left[{}^{\circ }\right]$ silanized / processed	${-}^{c}$	${-}^{c}$	$64\pm 7.7^{d}$	$81\pm 2.9^{d}$	$95\pm 2.0^{d}$	$84\pm 1.5^{d}$	$67\pm 2.6^{d}$
Fraction Octanol $X_{Oc}\left[\%\right]$ pristine	6.5±3.0	12±6.6	${-}^{e}$	${-}^{e}$	${-}^{e}$	${-}^{e}$	${-}^{e}$
Fraction Octanol $X_{Oc}\left[\%\right]$ silanized / processed	90±4.5	6.5±3.0	${-}^{e}$	${-}^{e}$	${-}^{e}$	${-}^{e}$	${-}^{e}$

${}^{a}$ Technical data sheet manufacturer |${}^{b}$ Measured with LS13 320 XR laser diffraction particle size distribution analyzer from Beckman Coulter Inc, USA. See Fig. SI.5 for full distribution |${}^{c}$ No comparable measurement for particle powders |${}^{d}$ Measured with the Dataphysics OCA-20 contact angle analyzer. Static contact angle measured by sessile drop method at $20^{\circ }C$ and 70% relative humidity with films of the same material by the same manufacturer. Ultrapure water with drop volume 10μL$|{}^{e}$ No reliable results due to accumulation in the liquid-liquid interface. All values are rounded to 2 significant digits

The zeta-potential of the particles reflects their surface charge and is thus an important parameter for assessing the aggregation behavior of particulate materials. However, measuring the zeta-potential for the investigated materials is challenging. The commonly applied electrophoretic technique, which is based on the diffusion-induced motion of the particles in the liquid, is only suitable for small particles (≲1μm) as the diffusive motion becomes negligible for larger particles. Alternatively, the streaming potential method is applicable to smooth surfaces, such as foils or films, but inappropriate for particulate materials. Therefore, data available in the literature are used to derive general trends for the zeta-potentials of the materials used in this study. AtpH=7, negative zeta-potentials ranging from−10mV to−40mV are reported for magnetite particles (Rhein et al., 2019b; Sun et al., 1998), cellulose (Cadena et al., 2009; Uetani and Yano, 2012), PET (Güney et al., 2015; Kirby and Hasselbrink Jr, 2004), PP (Slepička et al., 2010), LDPE (Kirby and Hasselbrink Jr, 2004), PVC (Güney et al., 2015; Kirby and Hasselbrink Jr, 2004; Rhein et al., 2019b) and PS (Kirby and Hasselbrink Jr, 2004). There is little information about the influence of silanization on the zeta potential of magnetite particles, however, only slightly altered zeta-potentials were reported for silanized SiO${}_{2}$ surfaces (Guo et al., 2008; Ji et al., 2017). Similarly, data on the effect of enzymatic and oxidative treatments of MP and cellulose on their zeta potentials are currently lacking. However, as a change in contact angle as shown in Table 1 generally results in more ionizable groups on the particle surface, it can be assumed that the absolute value of the zeta potential increases through extraction.

### 2.7 Gravimetric analysis

Gravimetric analyses were performed on an AX205 DeltaRange balance from Mettler Toledo, USA, with an accuracy of0.01mg. Prior to the separation experiments, the accuracy of the gravimetric analysis was assessed by spiking different masses ranging from5mg to40mg of PET to a50mL glass bottle. These suspensions were then filtered onto membranes (Whatman,25mm diameter,0.2μm pore size, Sigma-Aldrich) and dried at$40^{\circ }\;C$. The relative error of the gravimetric analysis was determined by comparing the mass of the membranes before and after filtration. The experiments were performed in triplicate. The relative error was almost constant over the various concentrations with an average value over all measurements of${\bar{\Delta }}_{rel}=6.2\%\pm 6.2\%$ (see SI.7).

### 2.8 Image analysis

The separated fraction in 3-component experiments were dispersed in octanol in an ultrasound bath for5min to break agglomerates between hydrophobic particles, subsequently filtered onto membrane filters (Whatman,47mm diameter,0.2μm pore size, Sigma-Aldrich) and dried at$40^{\circ }\;C$. Visible light microscopy images of these filters were recorded on a Nikon Eclipse Ni with an automated stage and using a Nikon PlanFluor 4x0.13 WD17.2 objective.

An area of$4500\times 4500\;{\mathrm{px}}^{2}$ corresponding to$\approx 32\times 32\;{\mathrm{mm}}^{2}$ (the largest square that can be fitted into the47mm filter) was selected for image analysis. The cellulose fibers in the images of the separated fraction were manually colored and then segmented to quantify the cellulose fibres based on binarized images. Cellulose was clearly distinguishable from the MP due to their characteristic, fibrous shape and the slightly different color. The binary images were processed using the open source software *Fiji* Schindelin et al. (2012) to determine the total 2D area of the cellulose fibers. As reference, the same initial amount of cellulose fibers as was used in the 3-component experiments (see Table 2), was filtered and processed as described above. As these samples contained cellulose fibers only, the identification of the cellulose fibers on the recorded images was automated using specific thresholds for segmenting the images. The separation efficiency of cellulose fibers was estimated by comparing the total area of cellulose on the filters resulting from separated samples to the respective area from the cellulose reference filters. The workflow of the image analysis pipleline is schematically shown in Fig. SI.7.

**Table 2:** Volume concentrations and ratios of the different particle types used in the 2- and 3-component experiments. MP: Microplastic particles. Corresponding masses of the individual particle types are provided in Table. SI.3.

	2-component	3-component
Magnetite [vol%]	$2.79\times 10^{-3}\pm 1.12\times 10^{-4}$	$2.78\times 10^{-3}\pm 1.60\times 10^{-4}$
MP, Cellulose [vol%]	$3.78\times 10^{-2}\pm 1.70\times 10^{-3}$	$1.94\times 10^{-2}\pm 1.18\times 10^{-3}$
Ratio MP, Cellulose/Magnetite [−]	13.54±0.61	6.96±0.42
Cellulose [vol%]	-	$1.90\times 10^{-2}\pm 1.16\times 10^{-3}$
Ratio Cellulose/Magnetite [−]	-	6.83±0.42

### 2.9 Experimental design

**Fig. 1:**
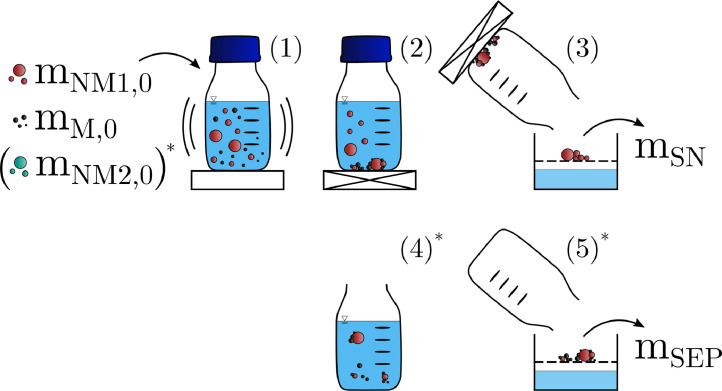
Schematic of the experimental procedure. $m_{M,0},m_{NM1,0},m_{NM2,0},m_{SN},m_{SEP}$: masses of the magnetic particles (M), the non magnetic particles (NM1,NM2: microplastic particles and/or cellulose), the particles in the supernatant (SN) and the separated fraction (SEP). ’0’ refers to the particles added at the beginning of the experiments. ${}^{*}$Only applied in 3-component experiments.

The experimental design is depicted in Fig. 1, with the experimental parameters listed in Table 2. Additional information about the absolute masses is given in Table. SI.2. Desired amounts of MP and/or cellulose particles were weighted into a50mL glass bottle (Schott AG, Germany) and magnetic particles into a separate weighting bowl. Then, ultrapure water (atrium pro, Sartorius, France) and a4M NaCl solution (analytical grade, EMSURE, Merck, Germany) were added to reach the liquid volume of40mL and the ionic strength (I) of the given experiment. ThepH was 7.05 (I=0.01M,$T=21.2^{\circ }C$). The magnetic particles were added to the MP suspension and the weighting bowl was rinsed with5mL of ultrapure water. Agglomeration between magnetic and non-magnetic particles was induced by putting the glass bottle for10min on a horizontal shaker (KS260 basic, IKA, Germany) at250rpm. Subsequently, the bottle was removed and placed next to a permanent magnet for2min to separate the magnetic agglomerates from the non-magnetic particles. The supernatant was carefully decanted while holding the permanent magnet in place.40mL of an NaCl solution with the same ionic strength as the respective experimental sample was added to rinse the glass bottle. A second magnetic separation was performed analogously but with a reduced magnetic separation time of1min. The collected supernatants were filtered onto a previously weighted membrane filter (Whatman,25mm diameter,0.2μm pore size, Sigma-Aldrich) and dried at$40^{\circ }\;C$ until constant weight, but at least for12h. In the 3-component experiments (MP, cellulose and magnetic particles), the separated fraction was resuspended, filtered and also weighted. This allowed the quantification of particle losses required for the calculation of the separation efficiencies. As this study was focused on the agglomeration behavior of the MP in the context of MP analysis, the possibilities to recycle the magnetic seed particles and ultimately the economy of the process were less relevant and thus not addressed in this work.

The separation efficiency for non-magnetic particles in the 2-component system$\left(T_{NM,2-comp}\right)$ was calculated according to Eq. 6. The assumption was that all magnetic particles added to the system$\left(m_{M,0}\right)$ were separated and therefore, particles in the supernatant$\left(m_{SN}\right)$ corresponded to not separated non-magnetic material at the end of the experiment ($m_{NM,E}=m_{SN}$) which was ensured by initial experiments. The initial mass of non-magnetic particles added to the system is denoted by$m_{NM,0}$.

(6) $$T_{NM,2-comp}=100\%\left(1-\frac{m_{NM,E}}{m_{NM,0}}\right)=100\%\left(1-\frac{m_{SN}}{m_{NM,0}}\right)$$

During 3-component experiments, the separated fraction was recovered and weighted to estimate particle losses during the procedure. The mass lossΔm over the individual 3-component experiments was calculated as the difference between the total mass of the particles recovered (mass of the supernatant$\left(m_{SN}\right)$ and of the separated fraction$\left(m_{SEP}\right)$) and the total mass of the added particles at the beginning of the experiment (MP$\left(m_{MP,0}\right)$, cellulose$\left(m_{Cellulose,0}\right)$, magnetite$\left(m_{magnetite,0}\right)$). Assuming that the particle losses can be equally distributed between the supernatant (SN) and separated (SEP) fraction and that cellulose is separated to the same degree as observed in the 2-component system ($T_{Cellulose,3-comp}=T_{Cellulose,2-comp}$), the separation efficiency of the MP in the 3-component system ($T_{MP,3-comp}$) was calculated according to Eq. 7. The validity of this assumption was assessed through microscopic image analysis of the separated fractions and is covered in Section 3.2.

(7) $$T_{MP,3-comp}=100\%\left(1-\frac{m_{SN}-\frac{\Delta m}{2}-m_{Cellulose,0}\left(1-\frac{T_{Cellulose,2-comp}}{100\%}\right)}{m_{MP,0}}\right)$$

## 3 Results & Discussion

### 3.1 2-Component System

**Fig. 2:**
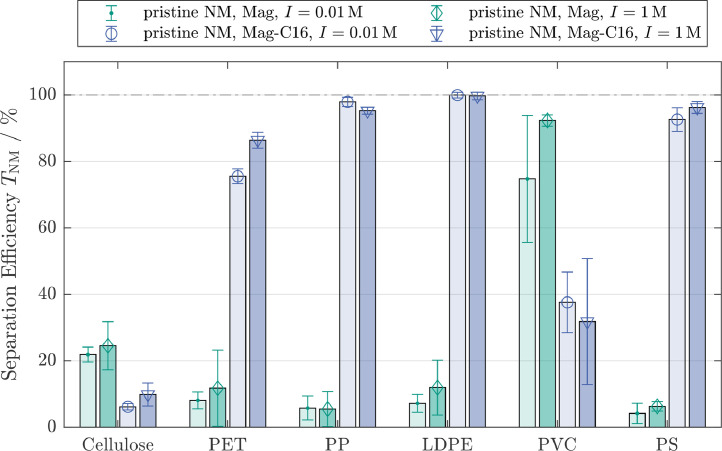
Separation efficiencies for pristine non-magnetic particle systems (NM: cellulose and different microplastic particle types) in the 2-component system. Green bars refer to experiments conducted with untreated magnetite (Mag) and blue bars refer to experiments conducted with hydrophobic magnetite (Mag-C16). The ionic strength was increased from 0.01M (left bars, light colors) to 1M (right bars, dark colors). PET: Polyethylene terephthalate, PP: polypropylene, LDPE: low-density polyethylene, PVC: polyvinyl chloride, PS: polystyrene)

The separation efficiencies when using untreated, hydrophilic magnetite (Mag) were around10% for PET, PP, LDPE and PS (Fig. 2) and cellulose was separated to≈20%. However, separation efficiencies exceeding80% were obtained for PVC. The separation behavior drastically changed, when using hydrophobic magnetic particles (Mag-C16). PP, LDPE and PS were almost completely separated and separation efficiencies of≈80% were obtained for PET. In contrast, the separation efficiencies of PVC and also for cellulose significantly decreased and were around30−40% for PVC and10% for cellulose.

These trends correlated with the hydrophobic properties of the respective polymer films listed in Table 1, except for PVC which is discussed separately. PP, LDPE and PS exhibit contact angles$>90^{\circ }$ and can be considered as hydrophobic (Israelachvili, 2011). The hydrophobic behavior of these polymers and Mag-C16 results in a strong attractive interaction between these particle types, enabling their agglomeration and subsequent magnetic separation. The origin of the hydrophobic force on a molecular level can be retraced to the rearrangement of water molecules between two hydrophobic surfaces (Israelachvili and Pashley, 1982), but hydrophobic particle-particle interactions are acting on a much larger length scale and are currently intensively debated. There seems to be a consensus that the origin of the long ranged attractive force is due to the bridging of nanobubbles attached on hydrophobic surfaces (Kékicheff, 2019), but Ishida et al. Ishida et al. (2018) demonstrated that even in the absence of such bubbles, strong attractive forces can be measured. When hydrophilic magnetic particles are used instead, the formation of hetero-aggregates is suppressed, which is reflected by the low separation efficiencies of the polymers. The separation efficiency for PET in combination with Mag-C16 was around80% and thus lower than the corresponding efficiencies for the PP, LDPE and PS. The slightly lower separation efficiency for PET may be explained by the lower contact angle of around$77^{\circ }$ resulting in a less efficient formation of hetero-aggregates.

The APSE results in Table 1 show that cellulose accumulates in the aqueous phase and can, thus, be considered as hydrophilic. The lower separation efficiencies when using Mag-C16 instead of hydrophilic magnetite can be explained by the formation of Mag-C16 homo-agglomerates. This reduces the total number of magnetic particles available to form hetero-agglomerates and reduces the aggregations kinetics (Eq. 1). This effect applies to all MP types, however, is offset for PET, PP, LDPE and PS by the additional attractive hydrophobic interaction leading to an increased collision efficiencyα.

Increasing the ionic strength by a factor of 100 from0.01M to1M did not significantly change the separation efficiency (Fig. 2), which is in contrast to previous studies on MSF (Rhein, Ruß, Nirschl, 2019, Rhein, Scholl, Nirschl, 2019, Rhein, Schmid, Esquivel, Nirschl, 2020). AtpH=7 all investigated particle systems are expected to be negatively charged (see Section 2.6), resulting in an electrostatic repulsion. According to SI.1 an increase in ionic strength reduces the range of the repulsive electrostatic interactions ($\kappa^{-1}↓$), lowers the energy barrier$E_{\Sigma ,max}$ and leads to an increased collision efficiencyα in Eq. 1. This facilitates the formation of aggregates and ultimately leads to an increase in the separation efficiency. The independence of the separation efficiencies from the ionic strength, therefore, suggests that the compression of the electric double layer did not change the aggregation behavior and that therefore, electrostatic forces were not controlling the hetero-aggregation process. Based on the results from previous studies Rhein, Ruß, Nirschl, 2019, Rhein, Schmid, Esquivel, Nirschl, 2020, Rhein, Scholl, Nirschl, 2019, only a negligible repulsion is expected atI=1M. The fact that this did not lead to an increase in the separation efficiency (in the experiments conducted with pristine magnetite), therefore indicates that the van der Waals attraction between particles is too weak to induce an agglomeration.

The most plausible explanation for this phenomenon is found in the particle diameters, which are orders of magnitude larger than in Rhein, Ruß, Nirschl, 2019, Rhein, Schmid, Esquivel, Nirschl, 2020, Rhein, Scholl, Nirschl, 2019: Whereas the van der Waals interaction increases proportionally to the particle radiusr, inertial forces or flow effects increase proportionally to the volume of the particle, i.e.$r^{3}$. A factor of 10 in diameter, consequently increases the ratio of inertial to van der Waals forces by a factor of 100. This effect is accounted for by the factor (a) in Eq. 3 and drastically reduces the collision efficiency for increasing particle diameter. Furthermore, the (cryo)-milling process likely increased the surface roughness of the polymers (see Fig. SI.2), which may lower their effective Hamaker constants by several orders of magnitude (Valmacco et al., 2016). The reduced van der Waals interactions thus explain the low separation efficiencies observed for PET, PP, LDPE and PS in combination with pristine magnetite. Similarly, cellulose exhibits diverse, fibrous shapes with rough surfaces, explaining the low van der Waals attraction and separation efficiency. Although hydrophobic interactions also increase proportionally to the particle radius (Christenson and Claesson, 2001), they nevertheless are able to induce an agglomeration despite the greatly increased inertial effects. Multiple studies (Christenson and Claesson, 2001; Rabinovich and Derjaguin, 1988) have shown that depending on the material system, the hydrophobic behavior can outperform van der Waals interactions by several orders of magnitudes and are therefore in agreement with the increased separation efficiencies of PET, PP, LDPE and PS when agglomerated with Mag-C16 as shown in Fig. 2.

The separation behavior of PVC was different to the other MP and substantially decreased from≈80% for pristine (hydrophilic) magnetite to≈35% for Mag-C16. This behavior is similar to the behavior observed for cellulose and generally indicates hydrophilic surface properties. Mag-C16 particles most likely homo-agglomerated during the experiment with PVC, which reduced the agglomeration kinetic (Eq. 1). The hydrophilic behavior of the PVC particles, however, is in apparent discrepancy to the results from the contact angle measurements of$\approx 86^{\circ }$ conducted on PVC films (Table 1), suggesting hydrophobic surfaces. Energy dispersive x-ray analyses conducted in the SEM (SEM-EDX) of a PVC film and of selected PVC particles revealed significantly different chlorine (Cl) contents (see SI.5), which indicate different surface properties of the particles compared to the films. Thus, the contact angle determined for the PVC films may not be transferable to the PVC particles. Based on the agglomeration and separation behavior of the PVC particleswe predict a lower contact angle of the PVC particles compared to the PVC films, consistent with the hydrophilic behavior of the PVC particles. Due the the experimental challenges outlined in Section 2.5, however, we were not able to experimentally assess the contact angle of the PVC particles. In addition to the surface properties, also the surface roughness of the PVC particles may have contributed to their hydrophilic behavior during hetero-agglomeration. In contrast to other MP, which were ground in a ball mill under cryogenic conditions, PVC was directly obtained in powder form and with a smaller particle size compared to the other MP (see Table 1 or Fig. SI.5). The high separation efficiencies for PVC of≈80% when pristine (hydrophilic) magnetic particles were used, suggest that van der Waals interactions exceeded inertial forces and were inducing the formation of agglomerates. This can be attributed to both the smaller particle size, reducing inertial forces, and larger effective Hamaker constants resulting from a smoother particle surface due to the manufacturing process of the PVC particles (see Fig. SI.2). Additionally, the experiments with PVC showed larger standard deviations, which may indicate weaker (van der Waals) contact forces in the agglomerates. Small variations in the experimental procedure may therefore result in agglomerate breakage and explain the variance in separation efficiency. Although it is difficult to quantify, differences in particle shape (see Fig. SI.2) may also influence the particle-particle collision and therefore the separation efficiency.

**Fig. 3:**
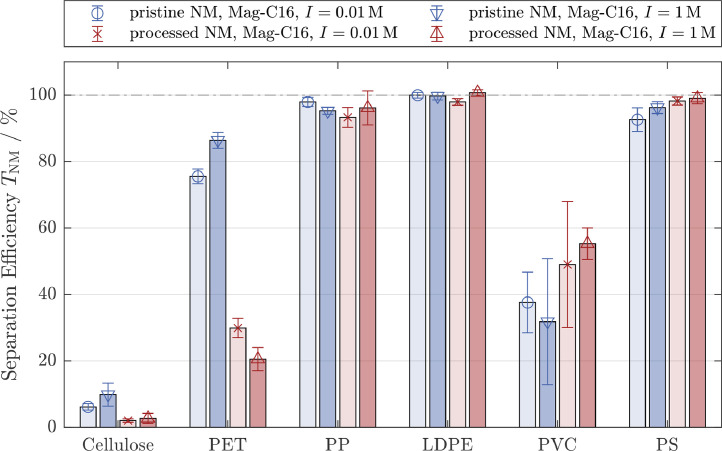
Separation efficiencies for pristine and processed non-magnetic particle systems (NM: cellulose and different microplastic particle types) in the 2-component system. All experiments were conducted with hydrophobic magnetite (Mag-C16). Blue bars refer to experiments conducted with pristine NM and red bars refer to experiments conducted with processed NM. The ionic strength was increased from 0.01M (left bars, light colors) to 1M (right bars, dark colors). Abbreviations for the different microplastic particle types are given in Fig. 2

All processed MP types, with the exception of PET, behaved very similar to the pristine ones during MSF (Fig. 3). Processed PP, LDPE and PS were almost quantitatively separated and processed cellulose was hardly separated at all. The average separation efficiency of PVC slightly increased to40−50%, but due to the large standard deviations, the effect is not considered significant. Table 1 further supports this, as the contact angle of PVC films remained unchanged through processing. The separation efficiency for processed PET, however, was reduced to20−30%. This reduction in separation efficiency can be explained by chemical changes modifying the surface properties of the PET which was reflected in a decreased contact angle of the processed PET of around$\approx 65^{\circ }$ (Table 1). The exposure of PET to strong oxidation agents (Fenton reagents) and to specific enzymes likely caused the formation of carboxylic acid end groups (Gewert et al., 2015)) rendering the surfaces more hydrophilic and explaining the change in the contact angle. The oxidation of PET surfaces is known to reduce the hydrophobicity i.e. increase the wettability and is a desired effect in designing membrane materials (Fávaro et al., 2007; Gotoh et al., 2018; Korolkov et al., 2015).

Although the contact angles of processed PP and PS were also reduced compared to the pristine materials, the separation efficiency for these polymers remained constant at close to100%. Thus, with the exception of PET, the separation efficiency of the pristine and the processed MP were similar, making the MSF a promising approach to isolate MP from digests of complex matrices (e.g. sewage sludge). Even for processed PET, the difference in the separation efficiency compared to cellulose is significant indicating a selective separation. By increasing e.g. agglomeration time or performing a multistage separation, the degree of separation for PET might be increased whereas cellulose is expected to remain in the suspension.

In summary, the results in Figs. 2 and 3 show that the separation efficiency is dominated by hydrophobic and van der Waals interactions. The ionic strength did not influence the separation efficiency, indicating that the electrostatic interactions were negligible for the investigated systems. Separation of hydrophobic nonmagnetic particles can be realized by using hydrophobic magnetic particles without increasing the separation efficiency of hydrophilic cellulose. Whereas the separation efficiencies for PP, LDPE and PS were mostly below 10% for hydrophilic magnetic particles, an almost complete separation was achieved with hydrophobic magnetite, independent of a previous processing steps. The different behavior of PVC suggests that the surface roughness plays a key role in MSF. Rough surfaces either created during the cryo milling process in the case of PET, PP, LDPE and PS or inherently present in the case of fibrous cellulose particles reduce the effective Hamaker constants which may help to explain the different separation behavior of these particles compared to PVC.

### 3.2 3-Component System

The results from the 2-component system suggested that a separation between hydrophobic MP and hydrophilic cellulose is possible. However, potential interactions between cellulose and MP may lead to an increase in the separation of cellulose and, thus, a reduced selectivity. A detailed discussion about selectivity in multi-component agglomerating systems is given in (Rhein et al., 2020). In the 3-component system, cellulose and MP can be present in the separated fractions. Therefore, more detailed information about the composition of these fractions in addition to gravimetic analyses were required to assess the separation efficiencies of the MP. In this study, the separated fractions of cellulose in each of the 3-component experiments were quantified by image analysis techniques as described in Section 2.8 and the obtained separation efficiencies of cellulose are shown in Table 3. Original and processed images are given in Fig. SI.8.

**Table 3:** Separation efficiency of cellulose in the 3-component system derived from image analysis of the separated fractions. Cellulose 1 and Cellulose 2 refer to different contrast thresholds applied to quantify the amount of cellulose on the reference image and thus represent min/max estimates. Abbreviations for the different microplastic particle (MP) types are given in Fig. 2

	Separation Efficiency Cellulose (3-component system) $T_{Cellulose,3-comp.}\left[\%\right]$				
MP type	PET	PP	LDPE	PVC	PS
Cellulose 1	0.85	0.38	0.58	1.2	0.37
Cellulose 2	1.6	0.72	1.1	2.3	0.69

**Fig. 4:**
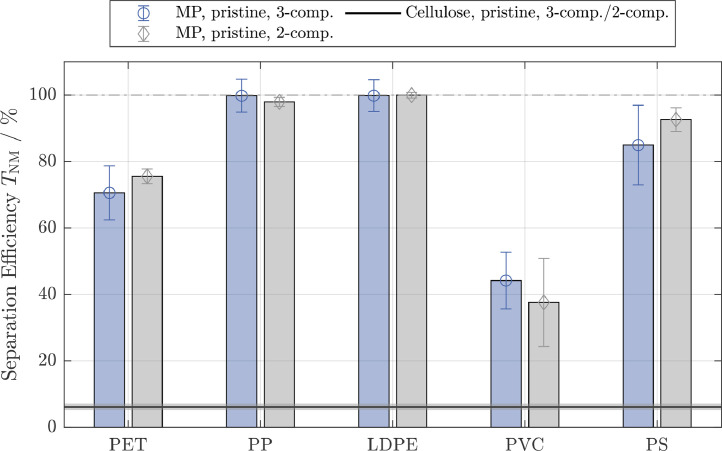
Separation efficiencies for different microplastic particle (MP) types in the presence of cellulose (3-component system). Experiments were conducted with pristine cellulose, pristine MP and hydrophobic magnetite (Mag-C16) (blue bars). The separation efficiencies from the respective experiments in the 2-component system (pristine MP and Mag-C16) are added for comparison (grey bars, see Fig. 3). The ionic strength was 0.01M and the separation efficiency for cellulose was assumed to be the same as in the 2-component system. Abbreviations for the different MP types are given in Fig. 2

**Fig. 5:**
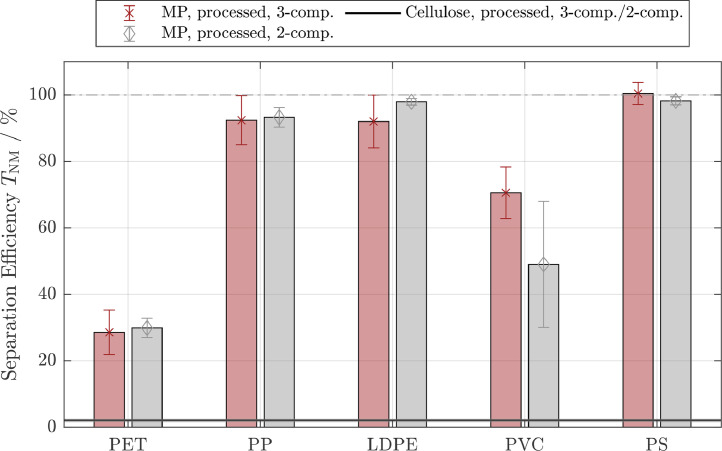
Separation efficiencies for different microplastic particle (MP) types in the presence of cellulose (3-component system). Experiments were conducted with processed cellulose, processed MP and hydrophobic magnetite (Mag-C16) (red bars). The separation efficiencies from the respective experiments in the 2-component system (processed MP and Mag-C16) are added for comparison (grey bars, see Fig. 3). The ionic strength was 0.01M and the separation efficiency for cellulose was assumed to be the same as in the 2-component system. Abbreviations for the different MP types are given in Fig. 2

The separation efficiencies for cellulose in all 3-component systems derived using image analysis tools, were in the range of0.37%−2.3%, indicating only a limited interaction between cellulose and the MP resulting in cellulose separation efficiencies similar to the 2-component system. Overlapping cellulose fibers, more prominent in the reference sample due to the higher cellulose concentration and manual identification of cellulose fibers in separated fractions both contributed to the uncertainty associated with the determination of separation efficiency in the 3-component system. We accounted for these effects by varying the image processing parameters (brightness/contrast settings for cellulose identification). As the separation efficiencies for cellulose obtained through image analysis were in good agreement with separation efficiencies of cellulose obtained from the 2-component systems, the separation efficiencies for cellulose in the 3-component system were set equal to the respective separation efficiencies determined in the 2-component system ($T_{Cellulose,3-comp.}=T_{Cellulose,2-comp.}$). The separation efficiencies of the MP were, thus, calculated according to Eq. 7 and are shown alongside the 2-component results in Fig. 4 for pristine and Fig. 5 for processed MP. The experiments were performed in suspensions with an ionic strength of 0.01 M and Mag-C16 particles. The cellulose separation efficiency is displayed with corresponding standard deviation as a horizontal line.

Within the standard deviations derived from three replicates, the pristine and processed MP showed an almost identical separation behavior in the 2- and in the 3-component system, which is consistent with the negligible interactions between cellulose fibers and MP. The results demonstrate that a selective separation between MP and cellulose with MSF is possible, and can reach high levels of selectivity. PP, LDPE and PS were quantitatively separated from the liquids, whereas cellulose was only separated to a limited extent. It is remarkable that the results from experiments conducted with PVC consistently show larger standard deviations compared to the results obtained from experiments conducted with the other MP. This may point towards the formation of weak hetero-agglomerates in the the secondary minimum, which may be ruptured to various extends during the magnetic separation process. The separation efficiencies for PET and PVC were around70% and40%, however, the selectivity of the process was still high. This means that PET and PVC will still be present in the supernatants, but the separated fraction will be almost cellulose-free. The overall separation might, therefore, be increased by extended agglomeration times or a multi-stage separation, resulting in higher recoveries in real-world samples, should this be necessary.

### 3.3 Separation of polymers from sewage sludge extracts

To assess the potential of the MSF approach to separate MP from digested sewage sludge extracts, additional separation experiments were performed. Digested sewage sludge samples were collected from a full-scale wastewater treatment plant (ARA Neugut, Duebendorf, Switzerland) and processed following the extraction protocol published by Philipp et al. (2022) and described in Section 2.4. The sludge extracts (EXS), essentially consisting of cellulose and polymers already present in the digested sewage sludge are considered as a separate type of nonmagnetic particle system. An initial separation experiment was performed with EXS yielding a separation efficiency of$T_{EXS}=16.65\;\%$, meaning that about17wt% of the particulate material present in the EXS was found in the separated fraction following the MSF approach and83wt% remained in the supernatant. Subsequently, processed PET (3.43mg±0.19 mg), PP (2.34mg±0.16 mg) and PVC (3.30mg±0.03 mg) were spiked to EXS (6.99mg±0.04 mg), resulting in identical volume concentrations of all MP and in similar volume concentrations of the sum of all MP and EXS. After the MSF of these experimental suspensions, both the absolute mass of particles in the supernatant$m_{SN,exp}$ and in the separated fraction$m_{SEP,exp}$ were determined gravimetrically. Assuming that the separation efficiencies determined for the 2- and the 3-component systems are also valid for multicomponent systems, the predicted mass of the supernatant ($m_{SN,pred}$) can be calculated according to Eq. 8

(8) $$m_{SN,pred}=\sum_{n=1}^{N}m_{n,0}\left(1-\frac{T_{n}}{100\%}\right)\;,$$

with$m_{n,0}$ referring to the initial masses of the individual, spiked MP and solids already present in the sludge extracts (EXS) and$T_{n}$ representing the separation efficiencies of the respective non-magnetic components (MP, EXS).

The mass of the particulate material in the supernatant, most likely dominated by the sum of spiked MP and MP/cellulose that were already in the sludge extracts, calculated by 8 was in good agreement with the experimental data shown in Table 4. Thus, the results suggest that MSF can be applied possibly as a final polishing step as part of a sample preparation protocol for the selective separation of MP from complex, environmental samples.

**Table 4:** Total initial mass of spiked microplastic particles (MP${}_{mix}$) and solids already present in the sludge extracts (EXS) $\sum_{n=1}^{N}m_{n,0}$, together with the experimental (measured) and the predicted total mass of the supernatants $m_{SN,exp}$ and $m_{SN,pred}$. Additionally, the difference between the measured and the predicted mass in the supernatants, expressed in % ($\Delta m_{SN}$), is provided. MP${}_{mix}$ 1-3 refer to three replicate experiments.

Sample	$\sum_{n=1}^{N}m_{n,0}\left[\mathrm{mg}\right]$	$m_{SN,exp}\left[\mathrm{mg}\right]$	$m_{SN,pred}\left[\mathrm{mg}\right]$	$\Delta m_{SN}\left[\%\right]$
EXS	6.86	5.72	-	-
EXS + MP${}_{mix}$ 1	15.77	9.95	9.94	+0.03
EXS + MP${}_{mix}$ 2	16.21	10.87	10.20	+6.16
EXS + MP${}_{mix}$ 3	16.17	9.49	10.04	−5.88

## 4 Conclusions

Cellulose fibers originating for example from disintegrating toilet papers, are abundant in digested sewage sludge. Recently published MP extraction protocols can efficiently isolate MP from most of the sludge components, but cannot separate MP from cellulose as MP and cellulose have similar densities and are both resistant against chemical and enzymatic digestions. For analytical purposes, e.g. pyrolysis GCMS measurements, the separation of cellulose materials from MP, however, would be highly welcome to avoid possible interference between pyrolysis products from MP and cellulose. This study demonstrated the potential of MSF using hydrophobic magnetic particles as seeding agents to separate MP from cellulose materials. For PP, LDPE and PS an almost complete separation of the MP from the liquids was achieved, whereas cellulose was only found in negligible amounts in the separated fractions. Although, PET and PVC were separated less efficiently from the liquids (≈75% and≈40%, see Fig. 4), the MSF was very specific as cellulose mostly remained in the supernatants. Therefore, the respective MP may be enriched in the separated fractions by repeated MSF or, alternatively, the obtained MP fractions may be corrected according to the respective separation efficiencies. The presented MSF approach using hydrophobic seed particles, represents a straight-forward and promising purification step, likely facilitating the quantification of MP extracted from complex matrices. The contrasting results obtained for PVC compared to other MP types emphasize the complexity of real-world MP systems and challenge the use of experimental results obtained from selected and / or specifically synthesized MP to assess the fate and behavior of MP in the environment.

## Declaration of Competing Interest

The authors declare that they have no known competing financial interests or personal relationships that could have appeared to influence the work reported in this paper.

## Appendix Supplementary materials

Image, application 1
