# Supplementary material for: Separation of Microplastic Particles from Sewage Sludge Extracts Using Magnetic Seeded Filtration
Source: Water Res X. 2022 Sep 13;17:100155. doi: 10.1016/j.wroa.2022.100155 (PMC9513278; doi:10.1016/j.wroa.2022.100155)
Supplement: Supplementary file 1 [file mmc1.pdf]

Supplementary Information:

Separation of Microplastic Particles from Sewage Sludge Extracts Using Magnetic Seeded Filtration

Frank Rhein[a], Hermann Nirschl[a], Ralf Kaegi[b]

[a] *Karlsruhe Institute of Technology (KIT), Institute of Mechanical Process Engineering and Mechanics Strasse am Forum 8, 76131 Karlsruhe (Germany), E-mail: frank.rhein@kit.edu*
[b] *Eawag, Ueberlandstrasse 133, 8600 Dübendorf (Switzerland)*

SI.1. Detailed information about particle-particle interactions

Fig. SI.1 shows exemplary interaction energy curves for the van der Waals (E_{vdW}), electrostatic (E_{el}) and hydrophobic (E_{hyd}) contributions, which add up to the sum curve (E_{Σ}). Approaching from large distances h and depending on the conditions, the sum curve may exhibit a secondary minimum (1), where particles are weakly bound to each other ($E_{\Sigma} < 0$). On further approach, the repulsive electrostatic interactions dominate, which leads to an increase in interaction energy and a repulsive force between the particles. For short distances, the van der Waals forces surpass the electrostatic forces and the particles agglomerate in the primary minimum (3). The energy barrier $E_{\Sigma,\mathrm{max}}$ (2) reflects the repulsive electrostatic forces separating the primary and secondary minimum. According to Eq. 3, the height of this energy barrier $E_{\Sigma,\mathrm{max}}$ governs the probability that two particles agglomerate upon collision. It is mainly affected by the electrostatic interactions, which are depending on the pH and ionic strength I of the suspension. The pH influences the surface charge of the particles and therefore the height of the energy barrier. The Debye length κ^{-1} is a measure for the diameter of the electrical double layer around the particles. It is proportional to $I^{-0.5}$ and I therefore governs the spatial extent of E_{el}. As is shown in Fig. SI.1, a decrease in I results in an increase in κ^{-1} and therefore an increased $E_{\Sigma,\mathrm{max}}$ reducing the collision efficiency α.

SI.2. Calculation of the volumes of silane required for the silanization of magnetite

Tab. SI.1 shows the estimated values of the surface specific silane number concentrations $N_{\mathrm{silane}}/A_{\mathrm{particle}}$ for three different studies. They are calculated from the reported mean particles diameter x_{50}, number concentration of silane $c_{\mathrm{n,silane}}$ and volumetric particle concentration $c_{\mathrm{v,particle}}$ according to Eq. SI.1. This assumes perfect spheres and neglects particle porosity (i.e. only the silanization on the outer surface is relevant).

$$\frac{N_{\mathrm{silane}}}{A_{\mathrm{particle}}} = \frac{c_{\mathrm{n,silane}}}{c_{\mathrm{v,particle}}} \frac{x_{50}}{6} \tag{SI.1}$$

As an excess of silane is not detrimental to the procedure, the highest value of 4×10^{-4} ([4]) is chosen. Eq. SI.1 can subsequently be rearranged to calculate the necessary concentration of silane for a given particles size and concentration.

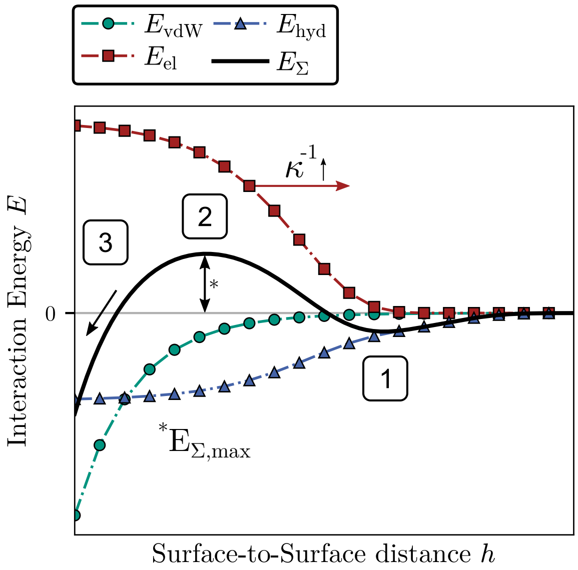

Figure SI.1: Schematic interaction energy curves for two particles approaching each other. The different energy contributions were calculated based on the following references: E_{vdw} [1], E_{el} [2] and E_{hyd} [3].

Table SI.1: Reported surface area specific concentration of silane required for the silanization process

Reference	Material	Estimated $N_{silane}/A_{particle}$ $\left[\mathrm{mol\,m^{-2}}\right]$
Ji et al. [5]	SiO_2	4.62×10^{-6}
Grbic et al. [4]	Fe	3.57×10^{-4}
Frickel et al. [6]	Fe_3O_4	3.84×10^{-5}

SI.3. Secondary electron images of the different particle types investigated in this study

Microplastic particles (MP), magnetite and cellulose particles were deposited on a sticky carbon tape for imaging in the SEM (Gemini 460, Zeiss, Germany). The microscope was operated at an acceleration voltage of 1 kV and 25 pA which allowed imaging the samples without a conductive coating. For image formation the secondary electron signal was used, in combination with an InLens detector. Selected images of the samples are shown in Fig. SI.2

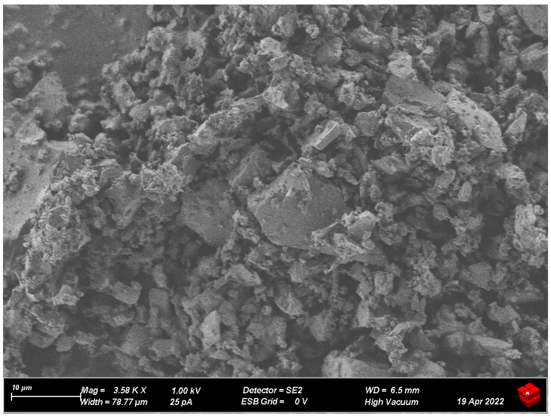

(a) Silanized magnetite (Mag-C16)

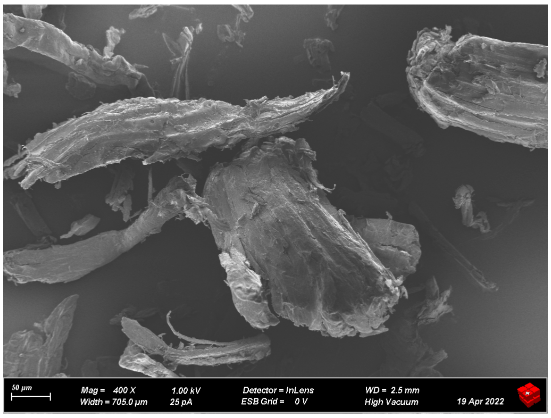

(b) Cellulose

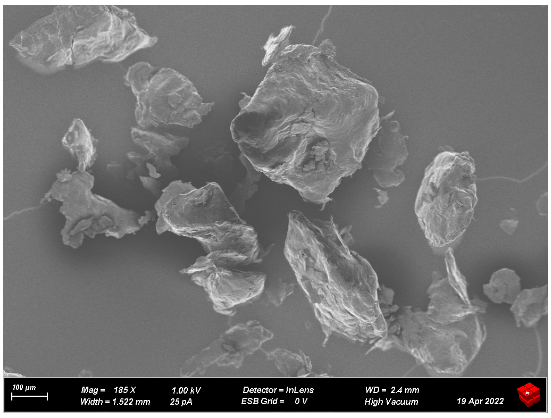

(c) Polyethylene terephthalate

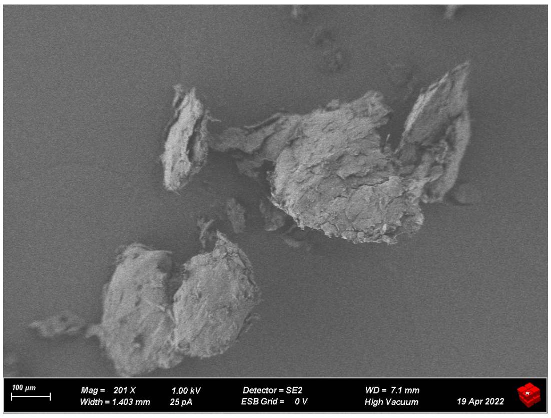

(d) Polypropylene

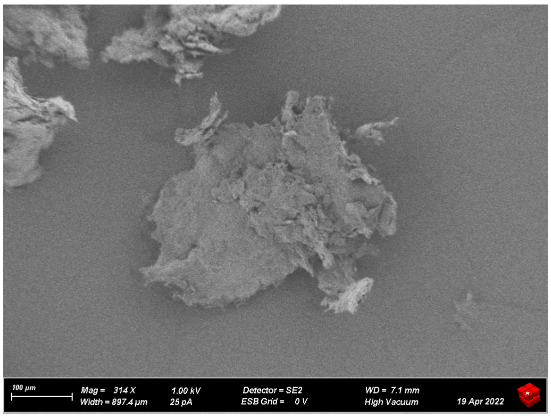

(e) Low density polyethylene

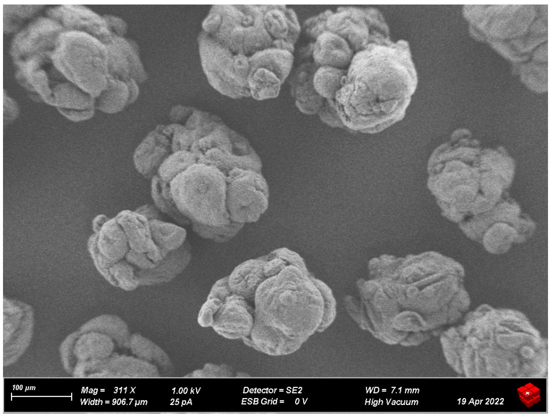

(f) Polyvinyl chloride

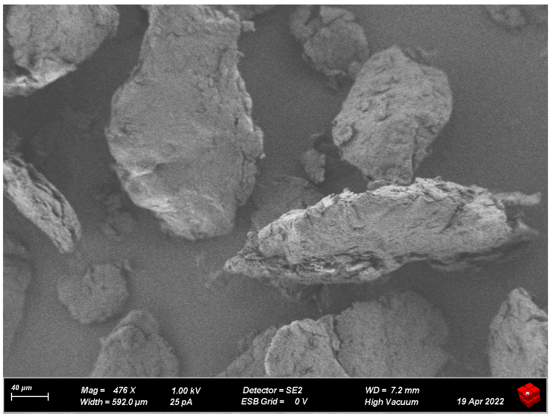

(g) Polystyrene

Figure SI.2: Secondary electron images of the different particle types investigated in this study.

SI.4. FTIR spectra of the different particle types investigated in this study

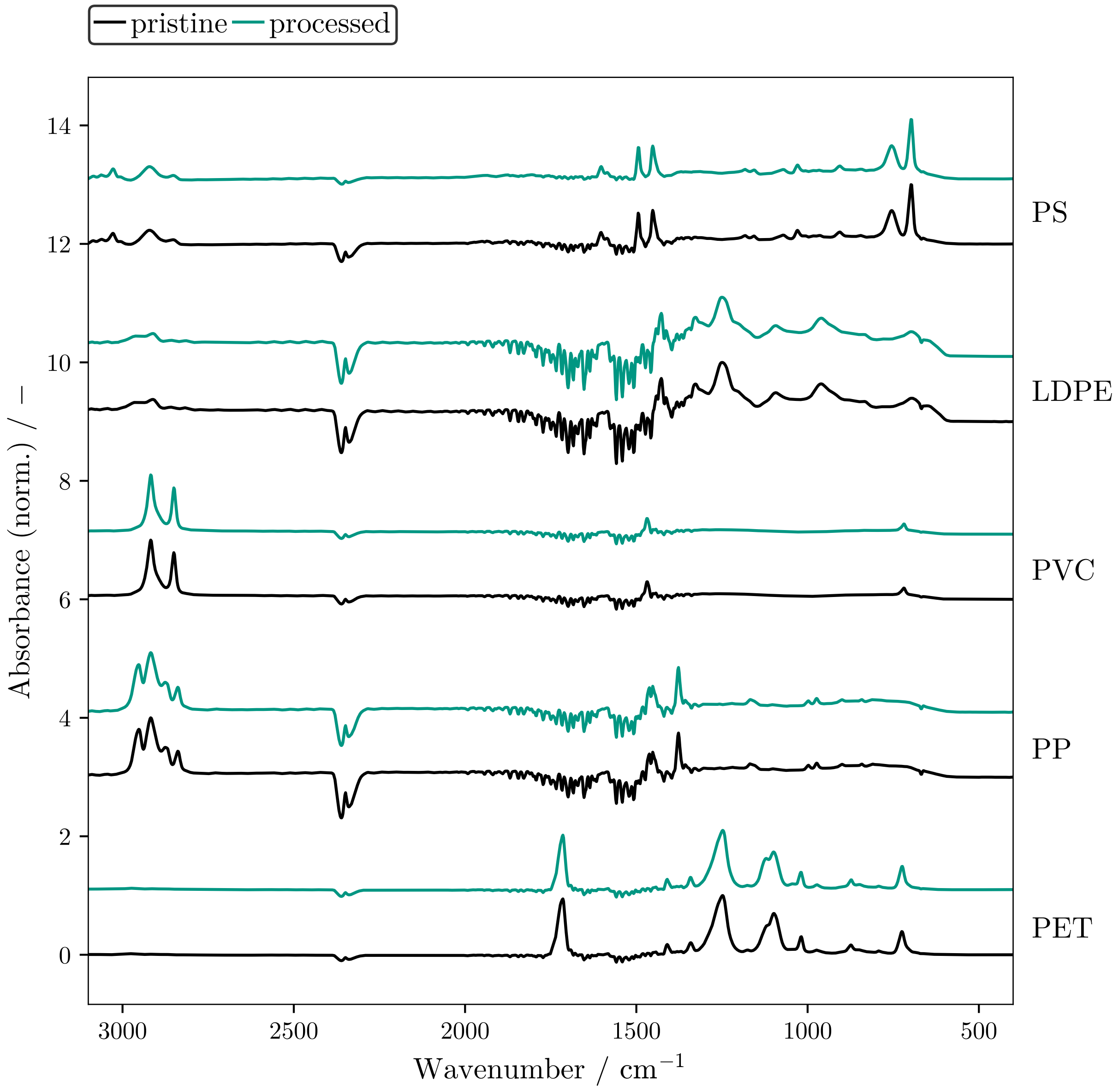

Figure SI.3: Attenuated total reflectance (ATR) Fourier Transform (FT) - Infrared (IR) spectra of pristine and processed polymers. The characteristic peaks of the polymers remained unaffected by the applied treatments (oxidative and enzymatic digestion). The oscillations at around 2300 cm^{-1} is caused by CO_2 and the oscillations at the sharp oscillations between around 1800 cm^{-1} and 1400 cm^{-1} are related to water vapor. The spectra were recorded on a a μ-FT-IR system (Cary 670 FT-IR instrument, Cary 610 IR microscope, Agilent) equipped with an ATR unit as described in [7]

SI.5. Elemental composition of polyvinyl chloride (PVC) particles and films

The elemental composition of PVC particles and PVC films was determined using an energy dispersive x-ray analysis system (Ultim Max detector 170 mm^2, Oxford Instruments, UK) attached to the scanning electron microscope (Gemini 460, Zeiss, Germany). For the elemental analyses the microscope was operated at an acceleration voltage of 3 keV. The results of the SEM-EDX measurement are shown together with theoretical elemental concentrations of PVC in Fig. SI.4.

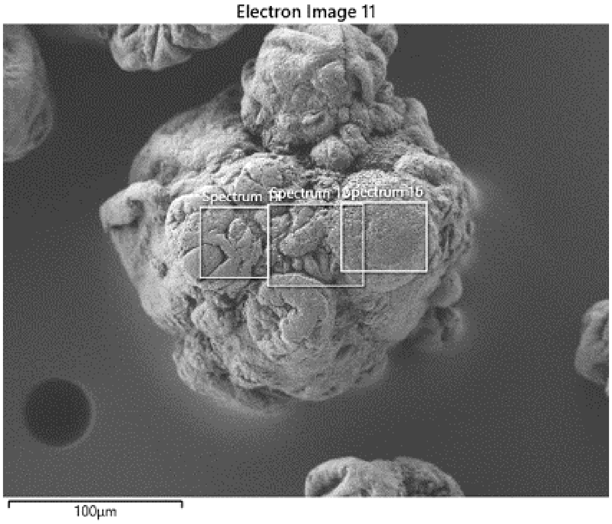

	C	Cl	O
Nominal	67%	33%	0%
Particles	85%	12%	2%
Films	69%	28%	4%

Figure SI.4: Secondary electron image of a polyvinyl chloride (PVC) particle (left) and elemental compositions of the PVC films and particles derived from SEM-EDX measurements (right). The nominal elemental composition of PVC is also provided.

SI.6. Particle size distributions

The cumulative particle size distributions of the relevant particle systems are shown in Fig. SI.5. They were measured with the LS13 320 XR laser diffraction particle size distribution analyzer from Beckman Coulter Inc, USA.

SI.7. Gravimetric analyses

Gravimetric analyses were performed on an AX205 DeltaRange balance from Mettler Toledo, USA, with an accuracy of 0.01 mg. The accuracy of the gravimetrical analysis was investigated by performing the entire experimental procedure discussed in section 2.9 without the addition of magnetic seed materials. Thus, all added non-magnetic particle mass is expected to be found on the membrane after filtration and drying. By comparing initial and final particle mass, the relative error of the gravimetric analysis was determined. The experiments were performed in triplicate with polyethylene terephthalate (PET) particles in four different initial concentrations and the detailed results are shown in Fig. SI.6.

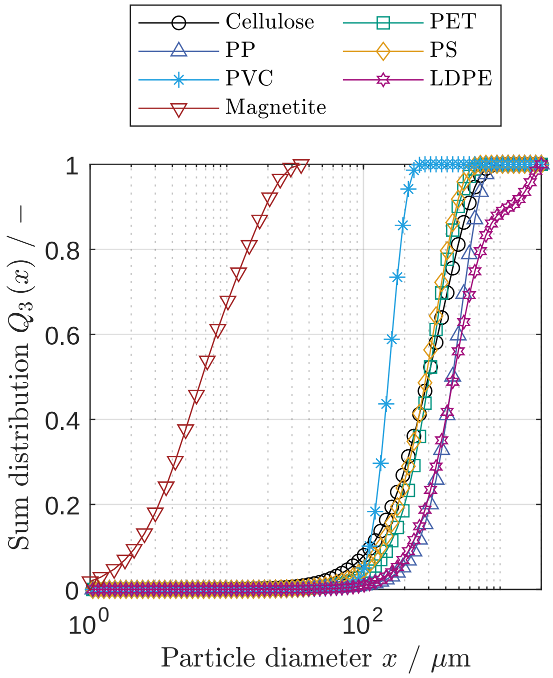

Figure SI.5: Cumulative particle size distributions of the investigated particle systems. PP: Polypropylene, PVC: Polyvinyl chloride, PET: Polyethylene terephthalate, PS: Polystyrene, LDPE: Low density polyethylene.

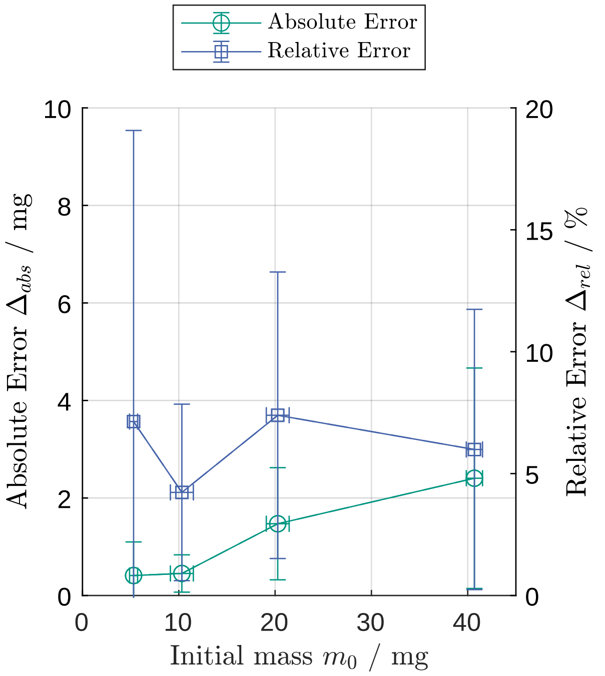

Figure SI.6: Absolute (Δabs) and relative (Δrel) error of the gravimetric analysis as a function of the initial mass.

SI.8. Workflow: Image analysis using Fiji

The image analysis workflow discussed in section 2.8 is shown graphically in Fig. SI.7 for the 3-component experiment in the [Mag-C16 | LDPE | Cellulose] system.

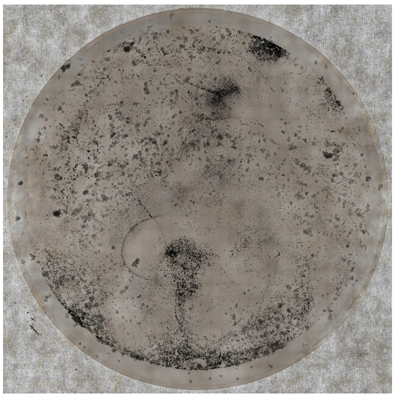

(a) Original image of the separated and filtered fraction [Mag-C16 | low density polyethylene (LDPE) | Cellulose].

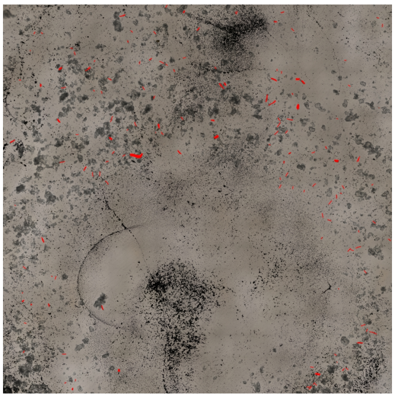

(b) Central part of the the Original image (a), with the cellulose manually marked in red ($4500 \times 4500\,\mathrm{px}^2$).

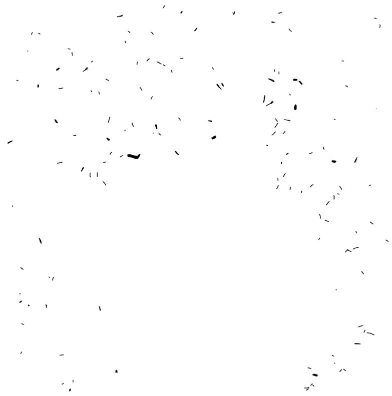

(c) Binary image of the central part (b), with the cellulose shown in black

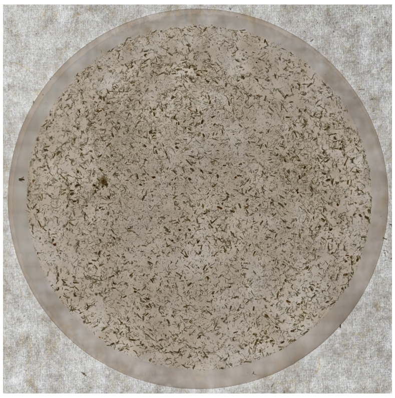

(d) Original image of the filtered cellulose reference sample. The same amount of cellulose was used as in the experiments described above, but instead of performing a separation experiment, the total amount of cellulose was deposited on the filters.

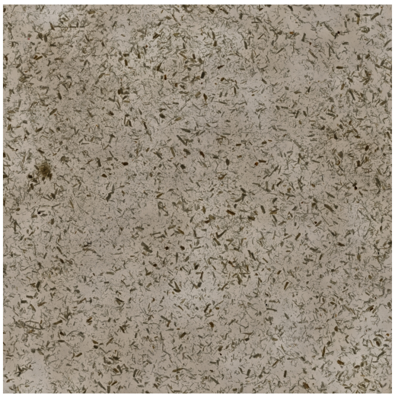

(e) Central part of the the original image (d) ($4500 \times 4500\,\mathrm{px}^2$).

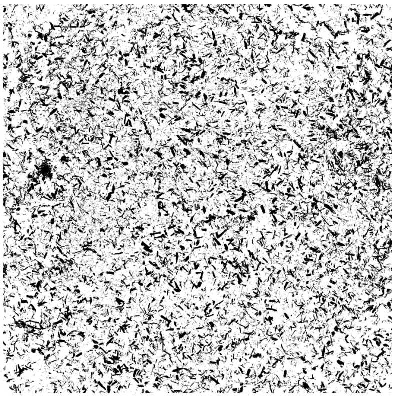

(f) Binary image of the central part (e), with the cellulose shown in black. Cellulose particles were automatically detected using an image threshold. These images were used as cellulose reference images for subsequent estimations of the separation efficiency of cellulose in the 3-component system.

Figure SI.7: Image processing and identification of the cellulose particles deposited on filters.

SI.9. Original and processed image data of the separated samples

The original and processed images of the separated samples of the 3-component experiments are shown in Fig. SI.8. Subsequent binarization and comparison to the cellulose reference image (same amount of cellulose deposited on a filter without magnetic separation, see Fig. SI.7f) yields an estimate of the separation efficiency of cellulose in the 3-component system ($T_{\mathrm{Cel},3-\mathrm{comp}}$, see Tab. 3).

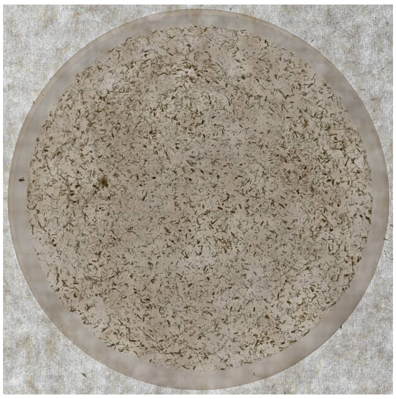

(a) Cellulose, reference image.

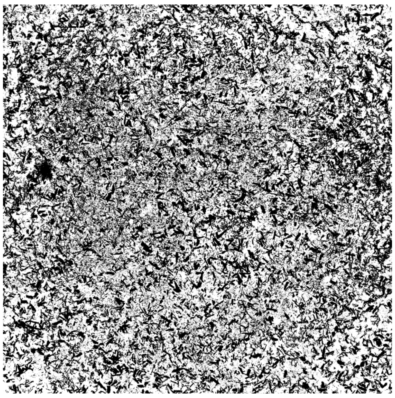

(b) Cellulose, binary reference 1 (low threshold, minimum estimate for $T_{\mathrm{Cel},3-\mathrm{comp}}$).

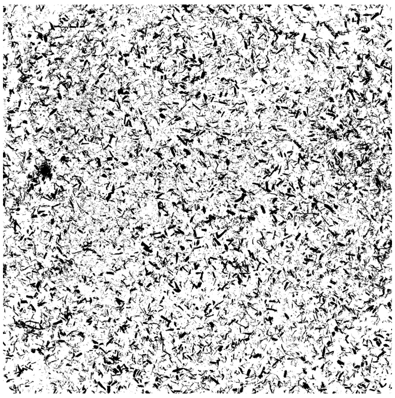

(c) Cellulose, binary reference 2 (high threshold, maximum estimate for $T_{\mathrm{Cel},3-\mathrm{comp}}$).

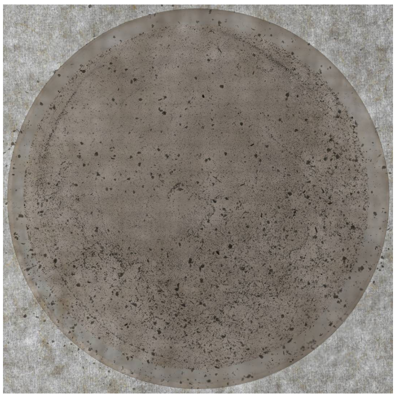

(d) PET, original.

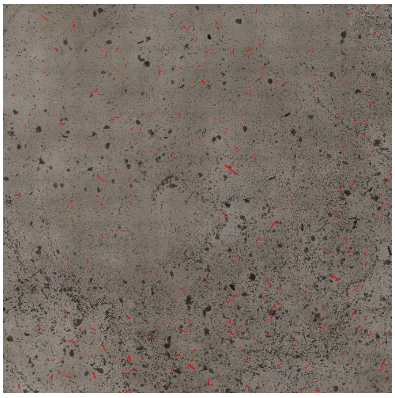

(e) PET, Cellulose colored.

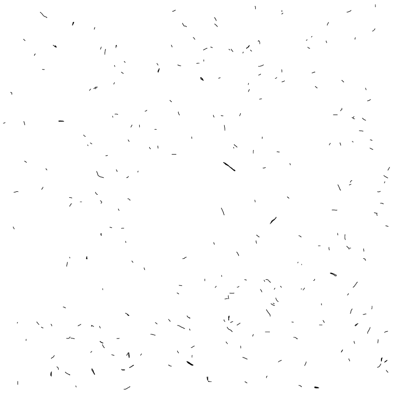

(f) PET, Cellulose binary.

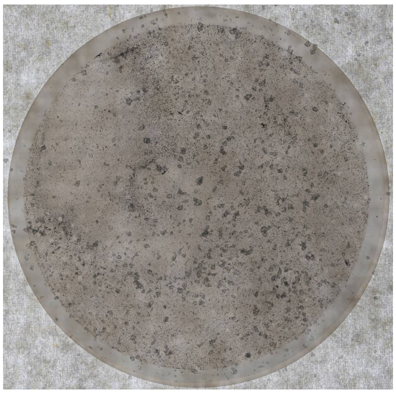

(g) PP, original.

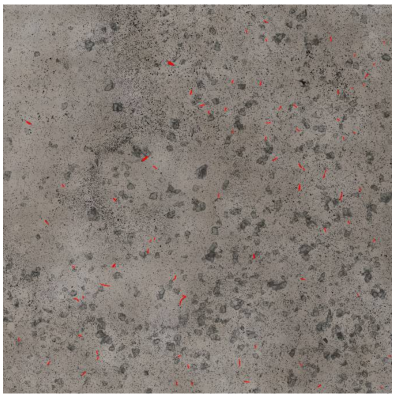

(h) PP, Cellulose colored.

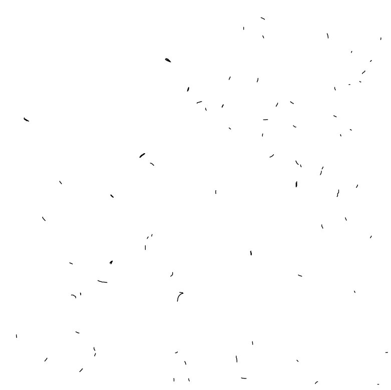

(i) PP, Cellulose binary.

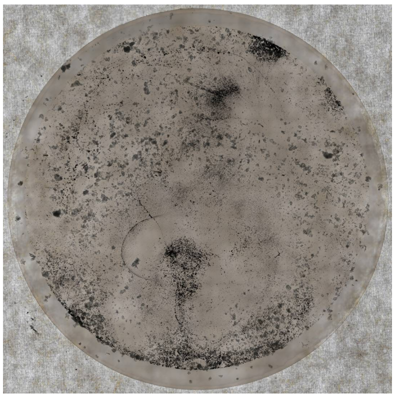

(i) LDPE, original.

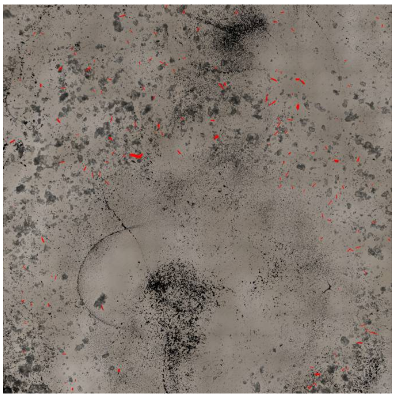

(j) LDPE, Cellulose colored.

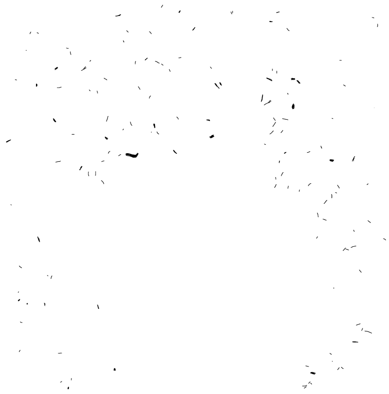

(k) LDPE, Cellulose binary.

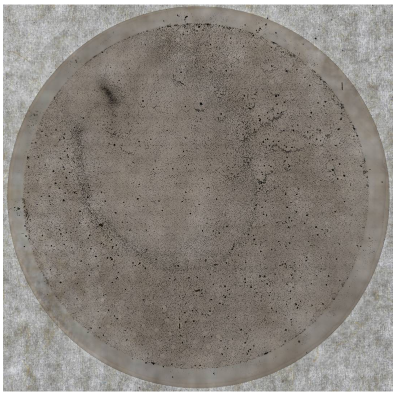

(l) PVC, original.

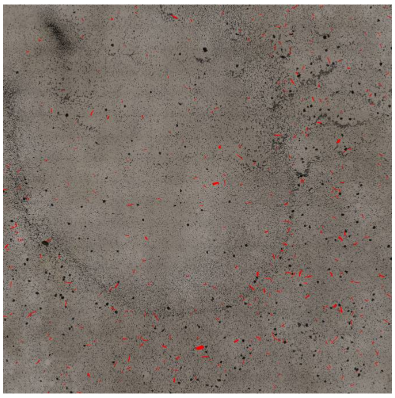

(m) PVC, Cellulose colored.

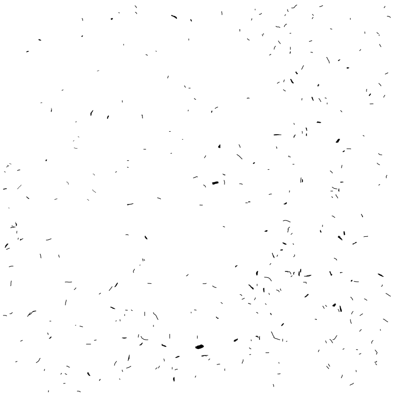

(n) PVC, Cellulose colored.

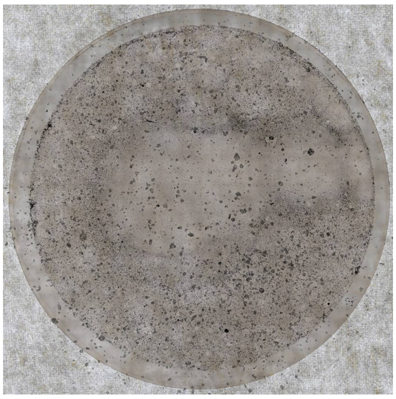

(o) PS, original.

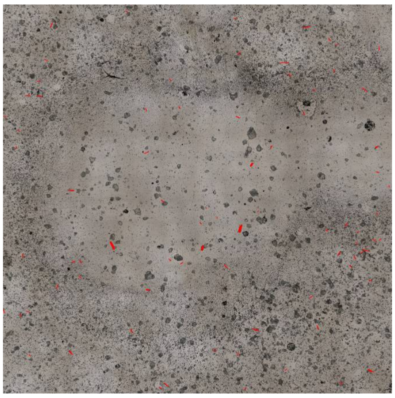

(p) PS, Cellulose colored.

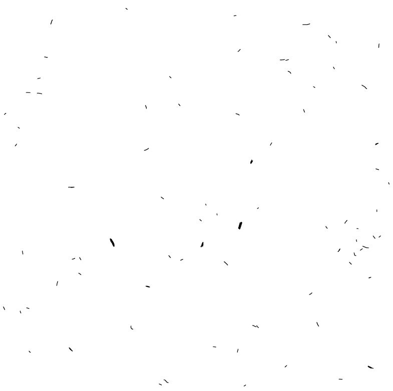

(q) PS, Cellulose colored.

Figure SI.8: Original and processed images of the separated fractions from experiments in the 3-component system. Abbreviations are given in Fig. SI.5.

SI.10. Total mass of particles used in the experiments

Table SI.2: Masses of the different particle types used in the 2- and 3-component systems. Abbreviations are given in Fig. SI.5.

Material	**2-component** m [mg]	**3-component** m [mg]
Magnetite	5.84 ± 0.05	–
Mag-C16	5.77 ± 0.30	5.79 ± 0.33
Cellulose	22.61 ± 0.41	11.37 ± 0.26
PET	20.83 ± 0.29	10.63 ± 0.28
PP	13.61 ± 0.30	6.92 ± 0.27
LDPE	14.01 ± 0.35	7.01 ± 0.33
PVC	20.9 ± 0.22	10.85 ± 0.66
PS	16.01 ± 0.31	8.28 ± 0.49

SI.11. Run-table of the experiments

Table SI.3: Run-table of the performed 2- and 3-component experiments in section 3.1 and 3.2. NM: non-magnetic particle type (cellulose / microplastic particles). Abbreviations are given in Fig. SI.5. Processed NM indicates whether or not the particles have undergone the oxidative and enzymatic treatment discussed in section 2.4.

No.	Magnetite [mg]	Mag-C16 [mg]	m_{NM1} [mg]	NM1	m_{NM2} [mg]	NM2	Processed NM	I [mol/L]
1	5.87	0	22.62	Cellulose	0	-	NO	0.01
2	5.91	0	22.31	Cellulose	0	-	NO	1
3	5.87	0	20.58	PET	0	-	NO	0.01
4	5.91	0	20.56	PET	0	-	NO	1
5	5.87	0	13.59	PP	0	-	NO	0.01
6	5.84	0	13.45	PP	0	-	NO	1
7	5.78	0	14.16	LDPE	0	-	NO	0.01
8	5.84	0	13.75	LDPE	0	-	NO	1
9	5.65	0	20.92	PVC	0	-	NO	0.01
10	6.01	0	20.78	PVC	0	-	NO	1
11	5.78	0	16.09	PS	0	-	NO	0.01
12	5.84	0	16.09	PS	0	-	NO	1
13	5.87	0	22.27	Cellulose	0	-	NO	0.01
14	5.95	0	22.51	Cellulose	0	-	NO	1
15	5.87	0	21.09	PET	0	-	NO	0.01
16	5.95	0	20.86	PET	0	-	NO	1
17	5.87	0	13.75	PP	0	-	NO	0.01
18	5.84	0	13.29	PP	0	-	NO	1
19	5.78	0	13.53	LDPE	0	-	NO	0.01
20	5.84	0	14.10	LDPE	0	-	NO	1
21	5.65	0	20.60	PVC	0	-	NO	0.01

22	6.01	0	20.69	PVC	0	-	NO	1
23	5.78	0	16.01	PS	0	-	NO	0.01
24	5.84	0	15.84	PS	0	-	NO	1
25	5.87	0	23.05	Cellulose	0	-	NO	0.01
26	5.95	0	22.94	Cellulose	0	-	NO	1
27	5.87	0	20.55	PET	0	-	NO	0.01
28	5.95	0	21.25	PET	0	-	NO	1
29	5.87	0	13.51	PP	0	-	NO	0.01
30	5.84	0	13.33	PP	0	-	NO	1
31	5.78	0	13.67	LDPE	0	-	NO	0.01
32	5.84	0	13.92	LDPE	0	-	NO	1
33	5.65	0	20.85	PVC	0	-	NO	0.01
34	6.01	0	21.56	PVC	0	-	NO	1
35	5.78	0	15.55	PS	0	-	NO	0.01
36	5.84	0	15.81	PS	0	-	NO	1
37	0	5.57	21.98	Cellulose	0	-	NO	0.01
38	0	5.72	23.00	Cellulose	0	-	NO	1
39	0	5.93	23.06	Cellulose	0	-	YES	0.01
40	0	5.70	22.66	Cellulose	0	-	YES	1
41	0	5.20	20.91	PET	0	-	NO	0.01
42	0	6.05	20.59	PET	0	-	NO	1
43	0	5.84	21.18	PET	0	-	YES	0.01
44	0	5.85	21.16	PET	0	-	YES	1
45	0	5.81	14.09	PP	0	-	NO	0.01
46	0	5.37	13.22	PP	0	-	NO	1
47	0	6.04	13.52	PP	0	-	YES	0.01
48	0	5.52	13.79	PP	0	-	YES	1
49	0	5.87	13.62	LDPE	0	-	NO	0.01
50	0	5.44	14.40	LDPE	0	-	NO	1
51	0	5.47	14.40	LDPE	0	-	YES	0.01
52	0	6.18	13.75	LDPE	0	-	YES	1
53	0	5.57	21.23	PVC	0	-	NO	0.01
54	0	5.41	21.06	PVC	0	-	NO	1
55	0	6.05	20.78	PVC	0	-	YES	0.01
56	0	5.88	20.61	PVC	0	-	YES	1
57	0	5.55	15.51	PS	0	-	NO	0.01
58	0	6.18	16.13	PS	0	-	NO	1
59	0	5.72	16.41	PS	0	-	YES	0.01
60	0	6.26	15.83	PS	0	-	YES	1
61	0	5.72	22.43	Cellulose	0	-	NO	0.01
62	0	5.27	22.05	Cellulose	0	-	NO	1
63	0	5.95	22.89	Cellulose	0	-	YES	0.01
64	0	6.11	23.16	Cellulose	0	-	YES	1
65	0	5.60	20.51	PET	0	-	NO	0.01
66	0	6.12	20.61	PET	0	-	NO	1
67	0	5.84	20.49	PET	0	-	YES	0.01
68	0	5.65	21.18	PET	0	-	YES	1
69	0	5.21	13.13	PP	0	-	NO	0.01
70	0	5.79	13.71	PP	0	-	NO	1

71	0	6.35	13.48	PP	0	-	YES	0.01
72	0	6.10	13.87	PP	0	-	YES	1
73	0	5.68	13.21	LDPE	0	-	NO	0.01
74	0	6.03	14.03	LDPE	0	-	NO	1
75	0	5.45	13.52	LDPE	0	-	YES	0.01
76	0	6.09	13.71	LDPE	0	-	YES	1
77	0	5.48	21.28	PVC	0	-	NO	0.01
78	0	5.55	21.07	PVC	0	-	NO	1
79	0	5.64	21.17	PVC	0	-	YES	0.01
80	0	5.78	21.05	PVC	0	-	YES	1
81	0	5.30	16.10	PS	0	-	NO	0.01
82	0	5.52	16.34	PS	0	-	NO	1
83	0	5.47	16.12	PS	0	-	YES	0.01
84	0	5.77	16.41	PS	0	-	YES	1
85	0	6.08	22.12	Cellulose	0	-	NO	0.01
86	0	6.28	22.21	Cellulose	0	-	NO	1
87	0	5.55	22.26	Cellulose	0	-	YES	0.01
88	0	5.89	22.15	Cellulose	0	-	YES	1
89	0	5.35	21.29	PET	0	-	NO	0.01
90	0	5.54	21.15	PET	0	-	NO	1
91	0	5.94	21.27	PET	0	-	YES	0.01
92	0	6.13	20.60	PET	0	-	YES	1
93	0	5.90	13.95	PP	0	-	NO	0.01
94	0	5.23	13.60	PP	0	-	NO	1
95	0	5.78	13.94	PP	0	-	YES	0.01
96	0	6.39	13.57	PP	0	-	YES	1
97	0	5.67	13.73	LDPE	0	-	NO	0.01
98	0	5.75	14.38	LDPE	0	-	NO	1
99	0	6.22	13.64	LDPE	0	-	YES	0.01
100	0	5.44	13.77	LDPE	0	-	YES	1
101	0	5.30	20.47	PVC	0	-	NO	0.01
102	0	5.64	21.71	PVC	0	-	NO	1
103	0	5.96	20.86	PVC	0	-	YES	0.01
104	0	5.83	20.77	PVC	0	-	YES	1
105	0	5.65	16.04	PS	0	-	NO	0.01
106	0	6.22	15.53	PS	0	-	NO	1
107	0	5.90	15.89	PS	0	-	YES	0.01
108	0	6.00	15.45	PS	0	-	YES	1
109	0	5.60	10.99	PET	11.78	Cellulose	NO	0.01
110	0	5.75	10.33	PET	10.89	Cellulose	NO	0.01
111	0	5.66	10.33	PET	11.53	Cellulose.	NO	0.01
112	0	5.27	10.36	PET	11.21	Cellulose	YES	0.01
113	0	5.29	10.95	PET	11.00	Cellulose	YES	0.01
114	0	5.69	10.58	PET	11.33	Cellulose	YES	0.01
115	0	6.16	7.26	PP	11.21	Cellulose	NO	0.01
116	0	5.44	7.15	PP	11.16	Cellulose	NO	0.01
117	0	5.52	6.52	PP	11.40	Cellulose	NO	0.01
118	0	5.47	6.69	PP	11.15	Cellulose	YES	0.01
119	0	5.48	7.12	PP	11.24	Cellulose	YES	0.01

120	0	6.52	7.13	PP	11.54	Cellulose	YES	0.01
121	0	5.44	6.51	LDPE	12.00	Cellulose	NO	0.01
122	0	5.74	7.20	LDPE	11.20	Cellulose	NO	0.01
123	0	6.18	7.36	LDPE	11.19	Cellulose	NO	0.01
124	0	5.64	7.43	LDPE	10.94	Cellulose	YES	0.01
125	0	5.76	6.63	LDPE	11.15	Cellulose	YES	0.01
126	0	5.62	7.42	LDPE	11.17	Cellulose	YES	0.01
127	0	6.54	10.99	PVC	11.01	Cellulose	NO	0.01
128	0	5.89	10.68	PVC	10.85	Cellulose	NO	0.01
129	0	5.80	10.11	PVC	11.34	Cellulose	NO	0.01
130	0	5.51	10.62	PVC	11.24	Cellulose	YES	0.01
131	0	6.41	12.45	PVC	11.82	Cellulose	YES	0.01
132	0	5.71	11.27	PVC	11.81	Cellulose	YES	0.01
133	0	6.07	8.54	PVC	11.19	Cellulose	NO	0.01
134	0	6.44	7.59	PVC	11.30	Cellulose	NO	0.01
135	0	5.82	7.80	PVC	11.10	Cellulose	NO	0.01
136	0	6.08	8.19	PVC	11.08	Cellulose	YES	0.01
137	0	5.39	7.71	PVC	11.44	Cellulose	YES	0.01
138	0	6.33	8.82	PVC	11.68	Cellulose	YES	0.01

References

[1] H. C. Hamaker, The London—van der Waals attraction between spherical particles, Physica 4 (10) (1937) 1058–1072. doi:https://doi.org/10.1016/S0031-8914(37)80203-7.

[2] J. Gregory, Interaction of unequal double layers at constant charge, Journal of Colloid and Interface Science 51 (1) (1975) 44–51. doi:https://doi.org/10.1016/0021-9797(75)90081-8.

[3] H. K. Christenson, P. M. Claesson, Direct measurements of the force between hydrophobic surfaces in water, Advances in Colloid and Interface Science 91 (3) (2001) 391–436. doi:https://doi.org/10.1016/S0001-8686(00)00036-1.

[4] J. Grbic, B. Nguyen, E. Guo, J. B. You, D. Sinton, C. M. Rochman, Magnetic extraction of microplastics from environmental samples, Environmental Science & Technology Letters 6 (2) (2019) 68–72. doi:10.1021/acs.estlett.8b00671.

[5] T. Ji, C. Ma, L. Brisbin, L. Mu, C. G. Robertson, Y. Dong, J. Zhu, Organosilane grafted silica: Quantitative correlation of microscopic surface characters and macroscopic surface properties, Applied Surface Science 399 (2017) 565–572. doi:https://doi.org/10.1016/j.apsusc.2016.11.241.

[6] N. Frickel, R. Messing, T. Gelbrich, A. M. Schmidt, Functional silanes as surface modifying primers for the preparation of highly stable and well-defined magnetic polymer hybrids, Langmuir 26 (4) (2010) 2839–2846. doi:10.1021/la902904f.

[7] M. Philipp, T. D. Bucheli, R. Kaegi, The use of surrogate standards as a QA/QC tool for routine analysis of microplastics in sewage sludge, Science of The Total Environment 835 (2022) 155485. doi:10.1016/j.scitotenv.2022.155485.
